# Supplementary material for: Effects of early-life antibiotics on the developing infant gut microbiome and resistome: a randomized trial
Source: Nat Commun. 2022 Feb 16;13:893. doi: 10.1038/s41467-022-28525-z (PMC8850541; doi:10.1038/s41467-022-28525-z)
Supplement: Supplementary file 1 — Supplementary Information [file 41467_2022_28525_MOESM1_ESM.pdf]

SUPPLEMENTARY INFORMATION

Reyman *et al.* Effects of early-life antibiotics on the developing infant gut microbiome and resistome: a randomized trial

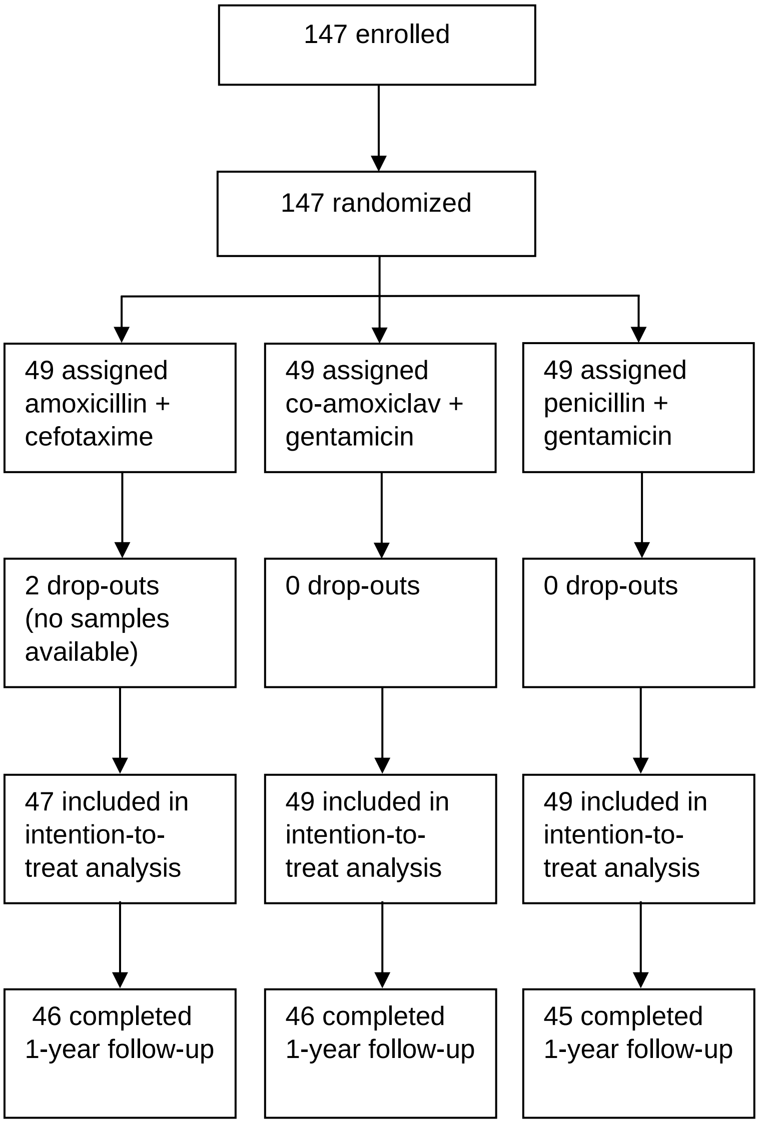

Supplementary Figure 1. Recruitment flowchart. Flowchart showing the number of children enrolled and finally included in the intention-to-treat analysis.

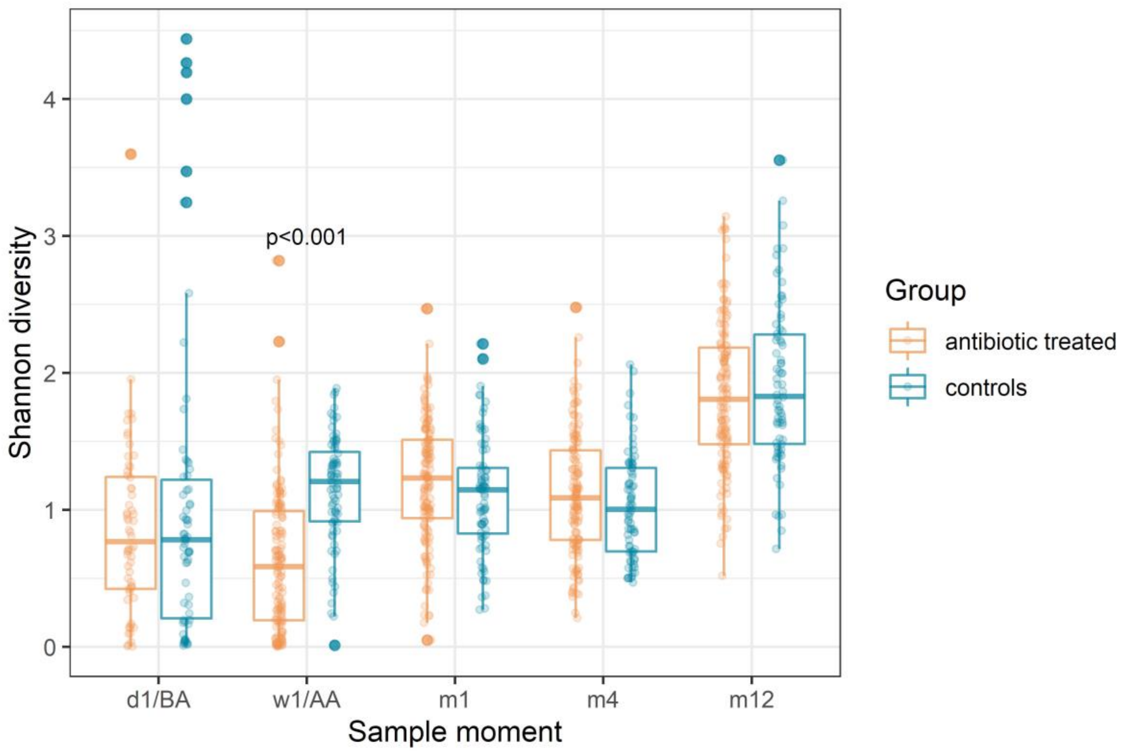

Supplementary Figure 2. α-diversity in antibiotic treated infants and controls. Differences in Shannon diversity of the faecal microbiota between antibiotic treated children and controls plotted per timepoint. Group differences were calculated using one-sided Wilcoxon tests. Boxplots with medians are shown; the lower and upper hinges correspond to the first and third quartiles (the 25th and 75th percentiles); the upper and lower whiskers extend from the hinge to the largest and smallest value no further than 1.5*IQR from the hinge; outliers are plotted individually by opaque circles; translucent circles visualize all data points. D = day, w = week, BA = before antibiotics, AA = after antibiotics, m = month. We observed a significant difference in Shannon diversity differed significantly between the antibiotic treated infants (orange, numbers for consecutive time points n=60, n=139, n=140, n=137 and n=133) and the controls (teal, n=66, n=80, n=80, n=78 and n=74) directly after antibiotic treatment (p=8.625e-14). Source data are provided as a Source Data file.

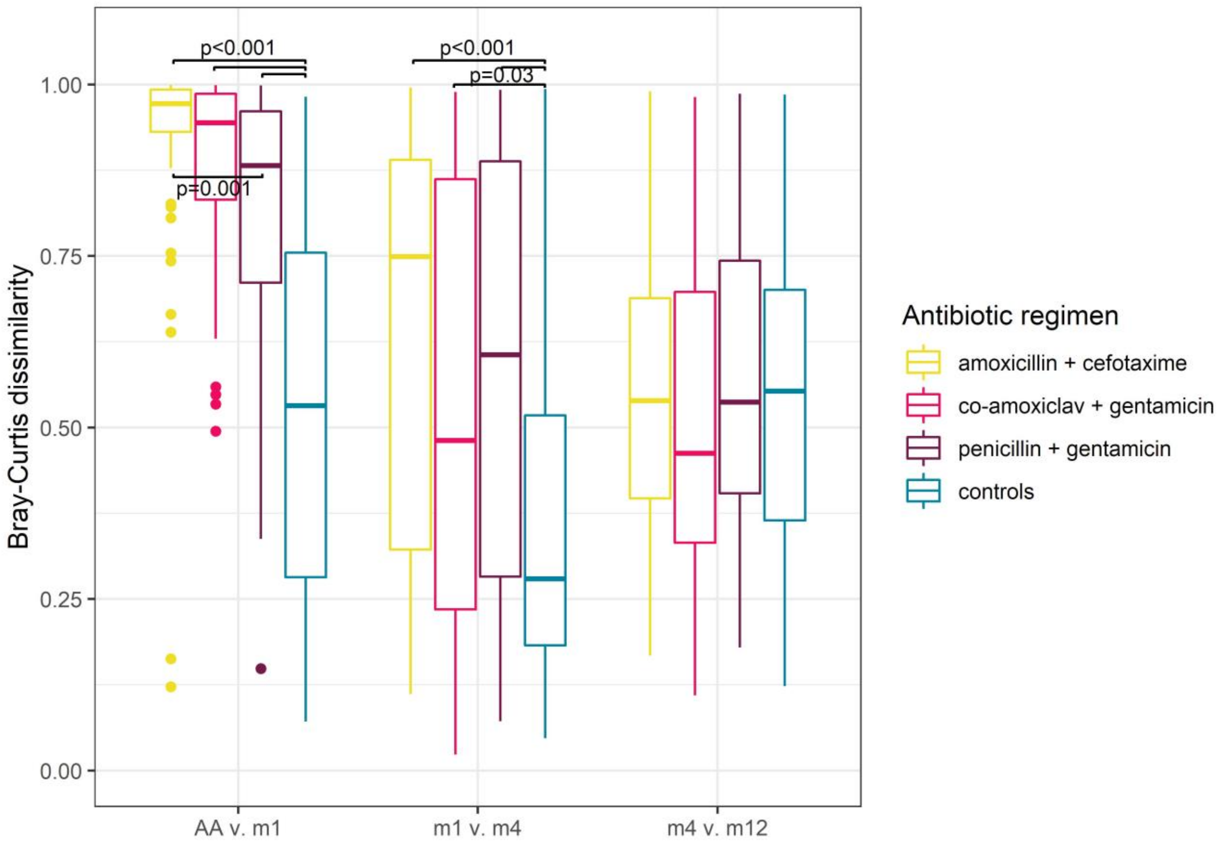

Supplementary Figure 3. Microbiota stability over time. As a measure of microbiota stability, we calculated the Bray-Curtis distance between consecutive sample pairs belonging to each individual per time interval, i.e. between stop of antibiotics and month 1, between month 1 and month 4, and between month 4 and month 12. Group differences were calculated using one-sided Wilcoxon tests. Boxplots with medians are shown; the lower and upper hinges correspond to the first and third quartiles (the 25th and 75th percentiles); the upper and lower whiskers extend from the hinge to the largest and smallest value no further than 1.5*IQR from the hinge; outliers are plotted individually. AA = after antibiotics, m = month. The datapoints of infants treated with amoxicillin + cefotaxime are colored yellow (n=45 for AA v. m1 comparison, n=45 for m1 v. m4 comparison and n=43 for m4 v. m12 comparison), co-amoxiclav + gentamicin are pink (n=44, n=46 and n=44), penicillin + gentamicin are purple (n=46, n=45 and n=44) and controls are teal (n=80, n=78 and n=72). In the comparison between stop of antibiotics and month 1: p=2.854e-13 for amoxicillin + cefotaxime vs. controls, p=5.968e-12 for co-amoxiclav + gentamicin vs. controls and p=8.158e-08 for penicillin + gentamicin vs. controls. In the comparison between month 1 and month 4: p=1.941e-05 for amoxicillin + cefotaxime vs. controls and p=0.000422 for penicillin + gentamicin vs. controls. Source data are provided as a Source Data file.

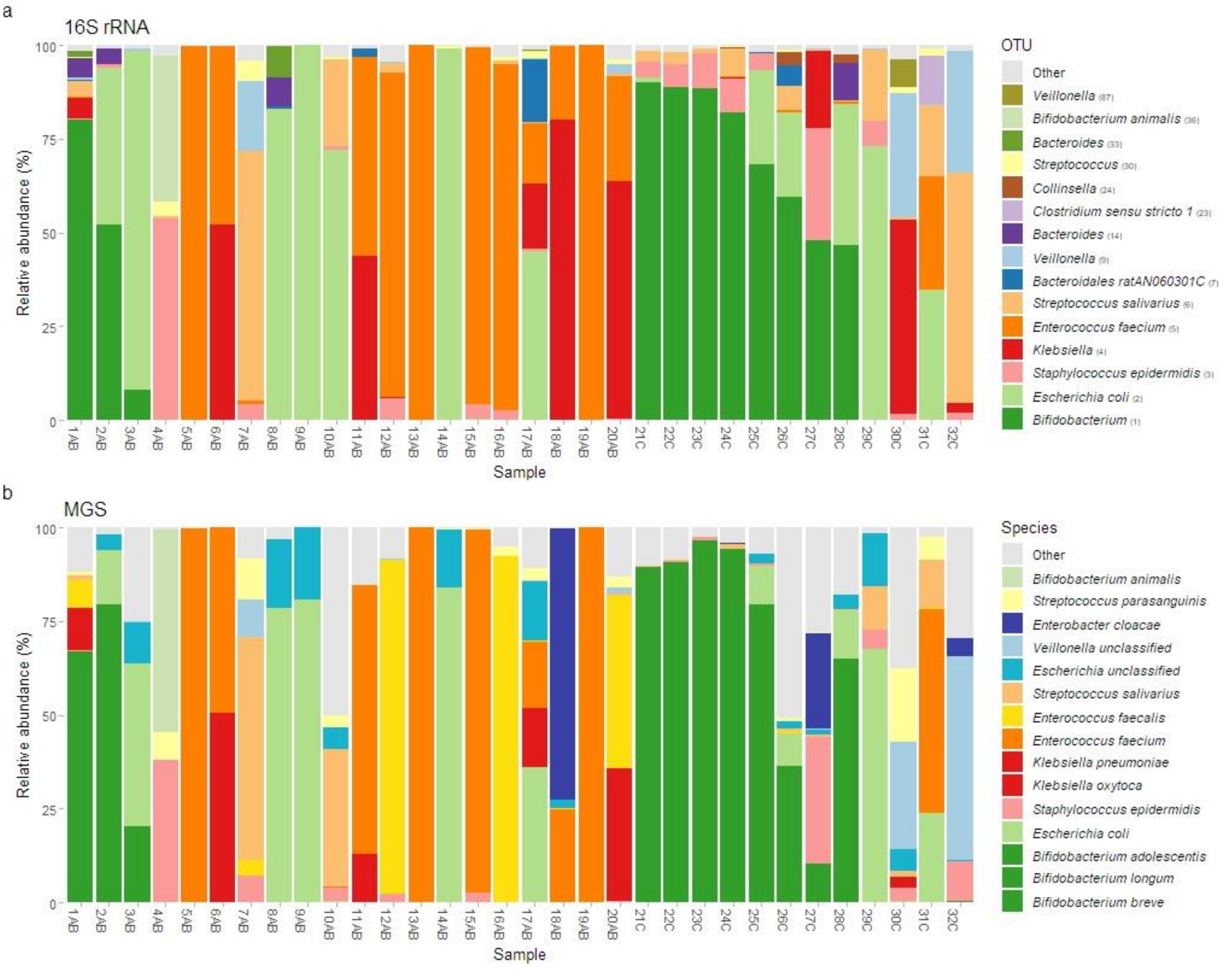

Supplementary Figure 4. Comparison of 16S rRNA OTUs with species found by MGS. A random subset of 32 samples was analysed by metagenomic shotgun sequencing (MGS). The mean relative abundances of the 15 most abundant 16S rRNA OTUs (**a**) were compared to the species found by MGS sequencing (**b**). Taxa approaching a similar annotation in both methods are coloured similarly to allow for easier visual comparison. AB = antibiotic treated, C = control. Source data are provided as a Source Data file.

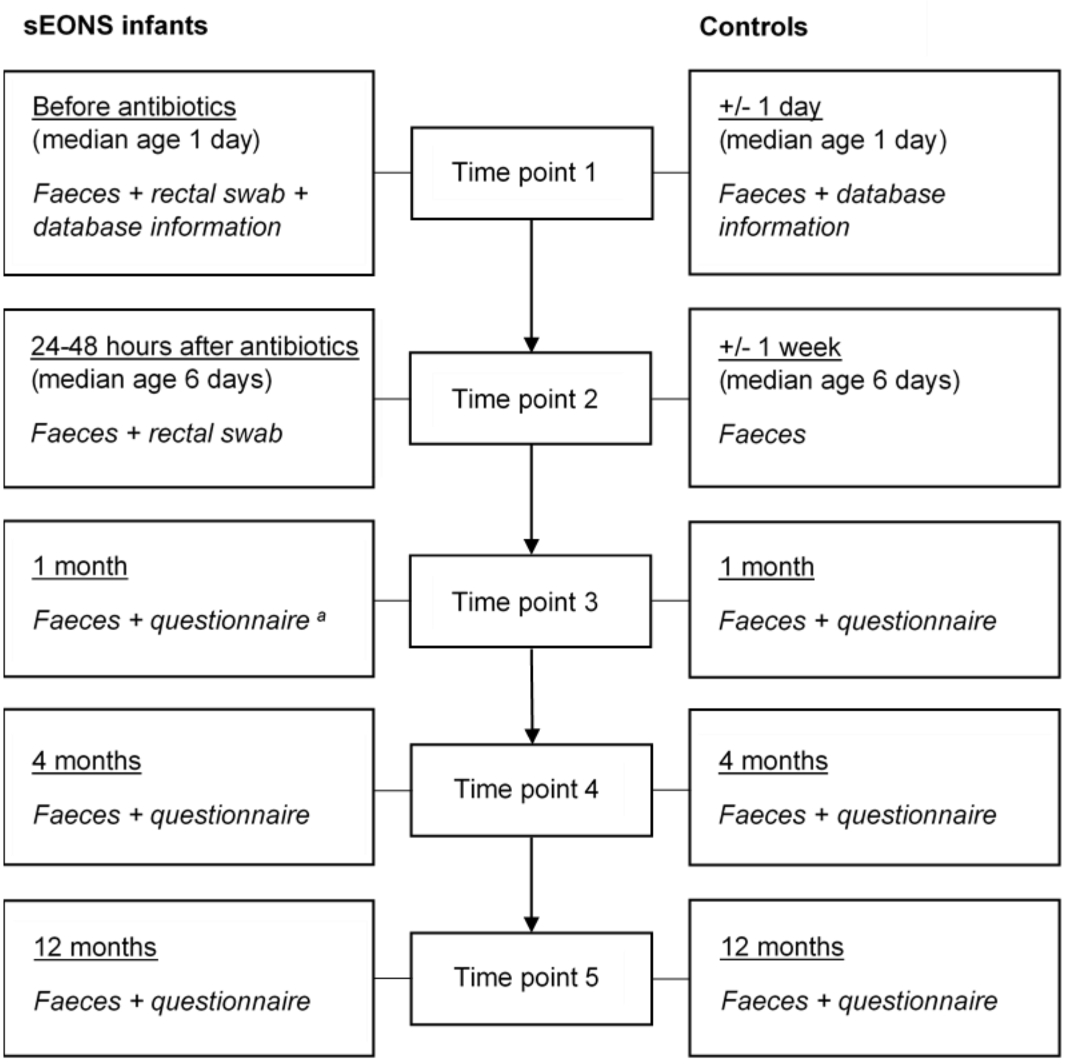

Supplementary Figure 5. Sample moments. [a]For the sEONS infants additional questionnaire information was collected at 6, 8 and 10 months of age; for the control infants this corresponded with questionnaires collected at 6 and 9 months of age.

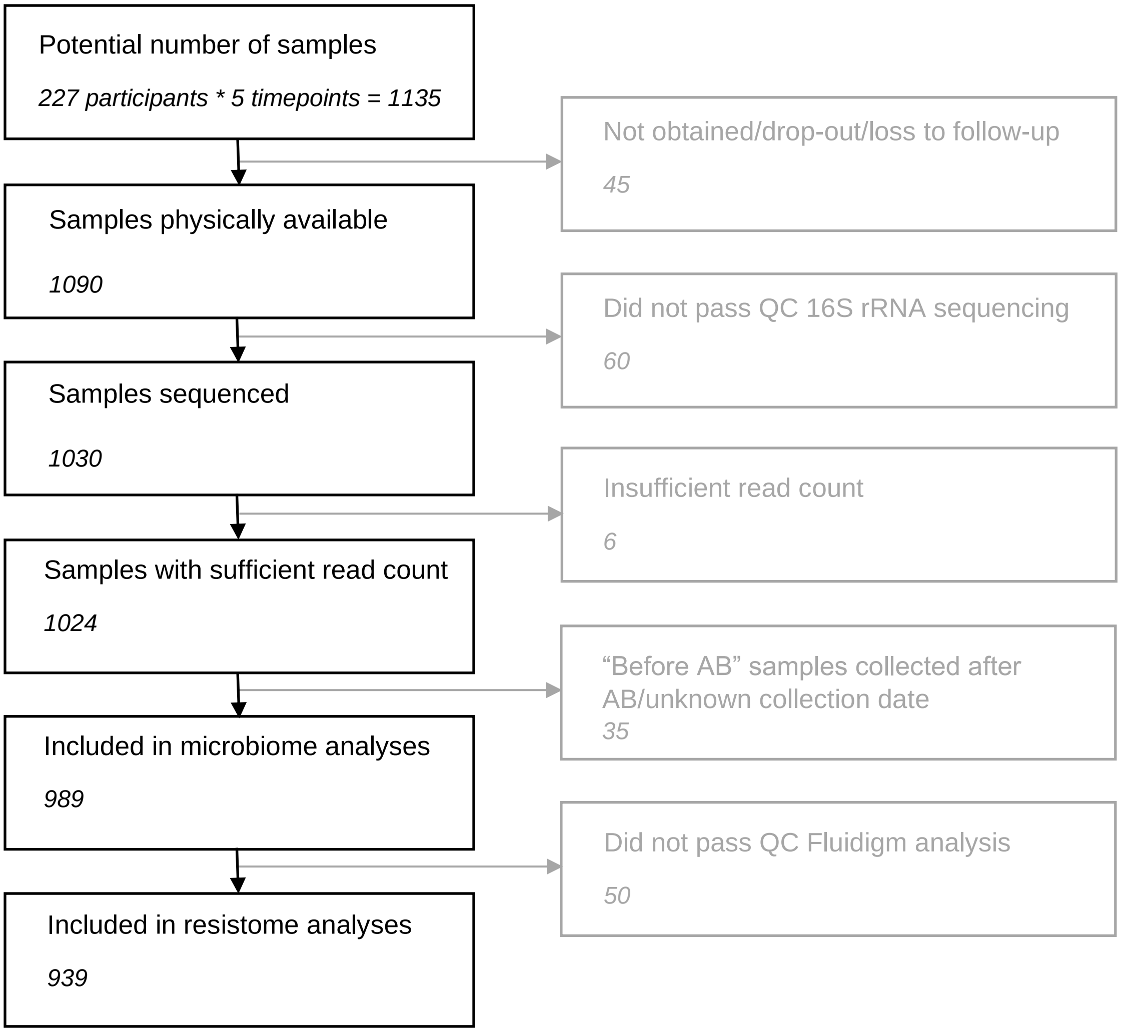

Supplementary Figure 6. Sample flowchart. Flowchart showing number of samples aimed for, obtained, and of sufficient quality for analyses. QC = Quality Control, AB = antibiotics.

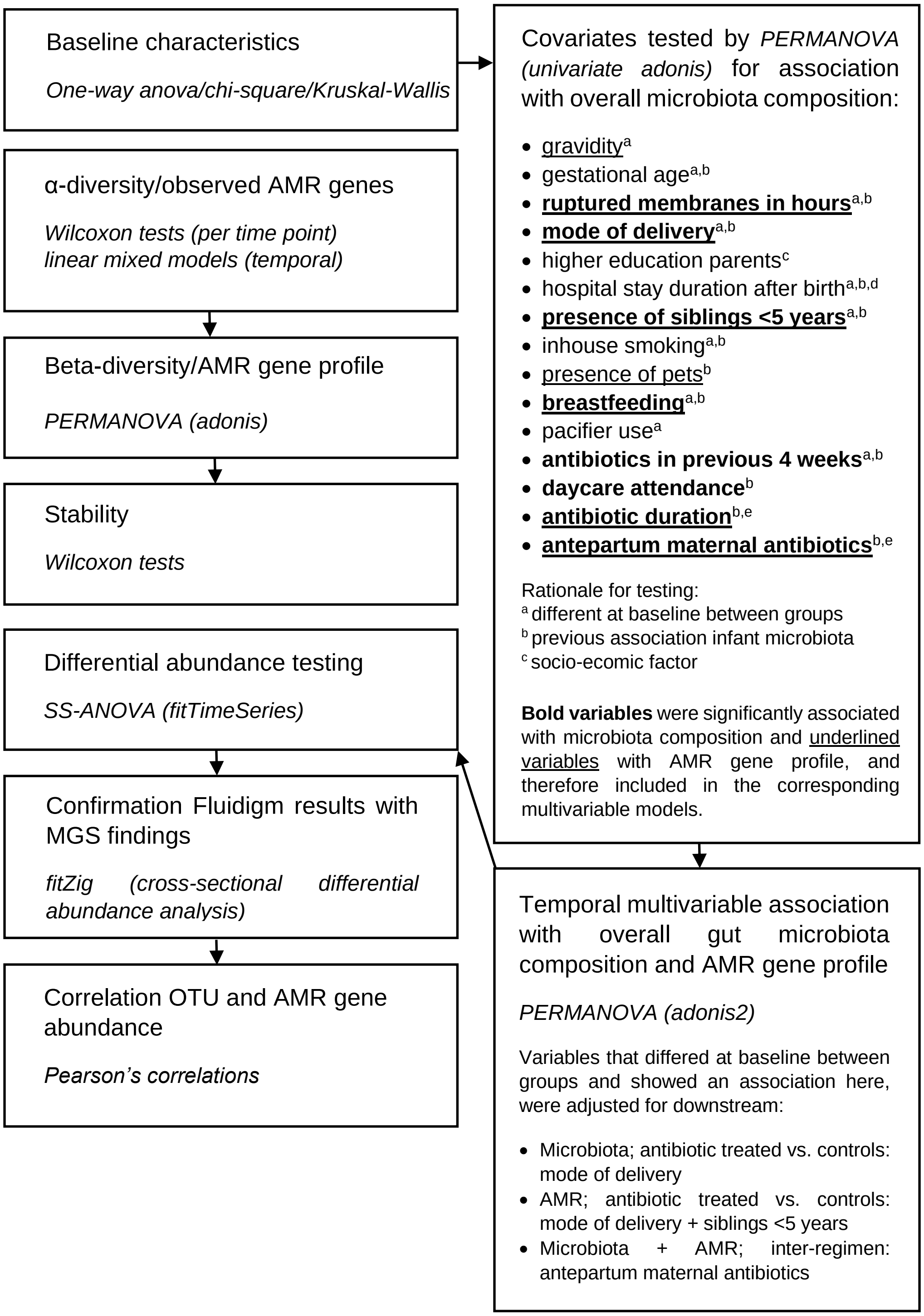

Supplementary Figure 7. Statistical analysis scheme. [d]Tested in stratified manner for the antibiotic treated infants and controls, because of collinearity with antibiotic treatment in the first week of life. [e]Variables only present in antibiotic treated infants. AMR = antimicrobial resistance, anova = analysis of variance, PERMANOVA = permutational multivariate ANOVA, SS-ANOVA = smoothing spline ANOVA, MGS = metagenomic shotgun sequencing, OTU = Operational Taxonomical Unit.

Supplementary Table 1: Baseline characteristics

	**Amoxicillin + cefotaxime**	**Co-amoxiclav + gentamicin**	**Penicillin + gentamicin**	**Controls**	***p***
n	47	49	49	80	
Gender, female (%)	22 (46.8)	26 (53.1)	19 (38.8)	43 (53.8)	0.36
Mode of delivery, secondary C-section (%)	6 (12.8)	10 (20.4)	15 (30.6)	8 (10.0)	*0.02*
Antepartum maternal antibiotics (%)	16 (34.0)	19 (38.8)	29 (59.2)	NA	*0.03*
Season of birth (%)					0.68
Winter	13 (27.7)	14 (28.6)	10 (20.4)	14 (17.5)	
Spring	8 (17.0)	6 (12.2)	8 (16.3)	21 (26.2)	
Summer	18 (38.3)	19 (38.8)	20 (40.8)	31 (38.8)	
Autumn	8 (17.0)	10 (20.4)	11 (22.4)	14 (17.5)	
Gravidity mothers, median (IQR)	1.00 (1.00, 2.00)	1.00 (1.00, 2.00)	1.00 (1.00, 2.00)	2.00 (1.00, 3.00)	*<0.001*
Gestational age in weeks, median (IQR)	40.29 (39.07, 40.93)	40.57 (39.86, 41.14)	40.43 (39.71, 41.29)	39.86 (39.11, 40.71)	*0.01*
Birth weight in grams, mean (SD)	3673.79 (623.99)	3632.41 (506.26)	3633.10 (628.74)	3494.91 (494.13)	0.27
Ruptured membranes in hours,<br>median (IQR)	8.50 (4.00, 21.00)	12.00 (6.00, 20.00)	11.00 (7.00, 17.50)	5.00 (2.50, 8.00)	*<0.001*
Apgar score at 5 minutes (%)					*0.008*
4	0 (0.0)	0 (0.0)	1 (2.0)	0 (0.0)	
5	0 (0.0)	0 (0.0)	2 (4.1)	0 (0.0)	
6	2 (4.3)	3 (6.2)	2 (4.1)	0 (0.0)	
7	4 (8.5)	4 (8.3)	4 (8.2)	2 (2.5)	
8	1 (2.1)	6 (12.5)	5 (10.2)	3 (3.8)	
9	5 (10.6)	13 (27.1)	8 (16.3)	10 (12.7)	
10	35 (74.5)	22 (45.8)	27 (55.1)	64 (81.0)	
Antibiotic treatment duration in days, median (IQR)	2.87 (2.38, 5.28)	4.50 (2.55, 6.57)	2.71 (2.29, 6.53)	NA	0.42

Antibiotics 1-4 days (vs. > 4 days) (%)	27 (57.4)	21 (43.8)	27 (55.1)	NA	0.36
Hospital stay duration in days, median (IQR)	5.00 (3.00, 7.00)	5.00 (3.00, 7.00)	5.00 (3.00, 7.00)	1.00 (1.00, 2.00)	*<0.001*
Number of siblings, median (IQR)	0.00 (0.00, 1.00)	0.00 (0.00, 1.00)	0.00 (0.00, 1.00)	1.00 (0.00, 1.00)	*0.001*
Presence of siblings <5 years of age (%)	11 (23.9)	11 (22.9)	12 (24.5)	43 (53.8)	*<0.001*
Pets (%)					0.35
None	24 (53.3)	24 (53.3)	34 (70.8)	44 (55.0)	
Cat(s)	14 (31.1)	14 (31.1)	10 (20.8)	17 (21.2)	
Dog(s)	4 (8.9)	1 (2.2)	2 (4.2)	7 (8.8)	
Cat(s) + dog(s)	0 (0.0)	0 (0.0)	0 (0.0)	2 (2.5)	
Other	3 (6.7)	6 (13.3)	2 (4.2)	10 (12.5)	
Inhouse smoking (%)	0 (0.0)	1 (2.1)	0 (0.0)	1 (1.2)	0.62
Parents finished higher education (%)	32 (71.1)	35 (74.5)	36 (73.5)	65 (81.2)	0.57
Breastfeeding in days, median (IQR)	37.50 (4.00, 153.00)	72.00 (11.75, 239.25)	94.00 (22.50, 350.50)	126.00 (4.50, 277.25)	0.08
Breastfeeding at 1 month (%)	25 (54.3)	32 (68.1)	33 (70.2)	55 (68.8)	0.32
Breastfeeding at 4 months (%)	13 (28.3)	18 (39.1)	23 (48.9)	41 (51.2)	0.07
Breastfeeding at 12 months (%)	2 (4.3)	9 (19.6)	12 (25.5)	12 (15.0)	0.05
Exclusive formula feeding (%)	11 (23.9)	8 (17.0)	7 (14.9)	12 (15.0)	0.59
Age start solid food in days, median (IQR)	142.50 (121.25, 159.50)	131.50 (120.25, 154.00)	135.00 (122.00, 159.00)	130.50 (119.00, 163.50)	0.82
Solid food at 4 months (%)	16 (34.8)	19 (41.3)	15 (31.9)	26 (32.5)	0.75
Pacifier use at 1 month of age (%)	37 (80.4)	33 (70.2)	35 (74.5)	57 (71.2)	0.65
Daycare since (%)					0.83
1 month	1 (2.2)	0 (0.0)	1 (2.2)	0 (0.0)	
4 months	20 (43.5)	20 (44.4)	22 (47.8)	35 (43.8)	
6 months	8 (17.4)	6 (13.3)	4 (8.7)	10 (12.5)	
10 months	6 (13.0)	1 (2.2)	2 (4.3)	10 (12.5)	

12 months	1 (2.2)	4 (8.9)	1 (2.2)	1 (1.2)	
>12 months	10 (21.7)	14 (31.1)	16 (34.8)	24 (30.0)	
Antibiotic use during follow-up (%)	10 (21.7)	7 (15.6)	5 (11.4)	21 (26.2)	0.20
Age at first antibiotic course during follow-up in days, mean (SD)	204.5 (82.3)	245.3 (78.3)	226.4 (61.7)	236.9 (89.0)	0.73
Antibiotics prior to 1 month time point (%)	1 (2.2)	0 (0.0)	0 (0.0)	1 (1.2)	0.64
Antibiotics prior to 4 months time point (%)	0 (0.0)	0 (0.0)	0 (0.0)	1 (1.3)	0.62
Antibiotics prior to 12 months time point (%)	9 (19.6)	7 (15.2)	5 (11.1)	20 (25.0)	0.27

Baseline characteristics stratified by antibiotic regimen. Categorical variables are shown in absolute numbers with percentages (%); continuous, normally distributed variables as means with standard deviations (SD); continuous, non-normally distributed variables as medians with interquartile ranges (IQR). One-way analysis of variance tests were used to compare means of normally distributed continuous variables; Kruskal-Wallis tests were applied to compare medians of non-normally distributed continuous variables; significant differences between categorical variables were tested with chi-square tests. The p-values of variables that differed significantly between the two groups are italicized for clarity. NA value means variable is not applicable to group (mothers of control infants did not receive antepartum antibiotics and control infants did not receive antibiotics in their first week of life, so this treatment duration is NA). Source data are provided as a Source Data file.

Supplementary Table 2: Subanalysis of effect of antibiotic treatment in the first week of life on overall gut microbiota composition in infants not receiving antibiotics later in life

Time point	R^2 (%)	Adjusted p-value
Before antibiotics	1.9	0.09
After antibiotics	11.2	0.001
Month 1	4.8	0.001
Month 4	1.5	0.05
Month 12	1.4	0.06

Effect sizes (R^2) and adjusted p-values (Benjamini-Hochberg method) are shown for the effect of antibiotics in the first week of life on overall gut microbiota composition of the subset of participants not receiving antibiotics later in life, as tested cross-sectionally per timepoint using a permutational multivariate analysis of variance (PERMANOVA) test. Source data are provided as a Source Data file.

Supplementary Table 3: Effect of short (1-4 days) versus long (>4 days) antibiotic treatment in the first week of life on overall microbiota composition

Time point	R^2 (%)	Adjusted p-value
Before antibiotics	1.3	0.63
After antibiotics	1.3	0.17
Month 1	1.3	0.17
Month 4	0.7	0.54
Month 12	1.3	0.17

Effect sizes (R^2) and adjusted p-values (Benjamini Hochberg method) are shown for the effect of short versus long antibiotic treatment in the first week of life on overall microbiota composition, as tested cross-sectionally per timepoint using a permutational multivariate analysis of variance (PERMANOVA) test. Source data are provided as a Source Data file.

Supplementary Table 4: FitTimeSeries results of all significantly differentially abundant taxa between antibiotic treated infants and controls

OTU	Int.no	Interval start	Interval end	Area	p.adj	ac	Ag	pg
Bifidobacterium_1	1	1	36	-285	0.0054	1-43	1-31	1-38
Bifidobacterium_1	2	119	123	-5	0.0054	NA	115-119	NA
Escherichia_coli_2	1	1	181	-236	0.0117	1-142	NA	1-177
Enterococcus_faecium_5	1	1	121	274	0.0054	1-117	1-106	1-119
Staphylococcus_epidermidis_3	1	1	229	-225	0.0206	12-175	1-29	1-211
Klebsiella_4	1	1	122	306	0.0161	16-127	1-256	1-106
Streptococcus_salivarius_subsp_thermophilus_6	1	1	30	-106	0.0054	1-27	1-30	1-27
Blautia_8	1	359	375	-22	0.0377	NA	*54-95	NA
Blautia_8	2	NA	NA	NA	NA	NA	neg 126-162	NA
Blautia_8	3	NA	NA	NA	NA	NA	neg 358-411	NA
ratAN060301C_7	1	1	388	-712	0.008	1-343	1-310	NA
Ruminococcus_gnavus_CC55_001C_10	1	1	195	-309	0.0117	1-64	1-151	1-238
Streptococcus_11	1	1	411	768	0.0195	1-384	1-411	1-403
Bifidobacterium_12	1	1	19	-51	0.0054	1-18	1-17	1-19
Bifidobacterium_12	2	35	88	133	0.0311	NA	NA	NA
Bifidobacterium_bifidum_NCIMB_41171_13	1	1	39	-149	0.0054	1-213	1-34	1-43
Bifidobacterium_bifidum_NCIMB_41171_13	2	97	128	-94	0.0054	NA	99-121	NA
Veillonella_9	1	2	15	-62	0.0054	4-17	5-18	1-21
Veillonella_9	2	117	128	25	0.0054	119-127	118-127	118-123
Bacteroides_14	1	1	255	-425	0.0117	NA	1-244	1-169
Clostridium_sensu_stricto_1_15	1	1	10	-20	0.008	1-10	1-10	1-2
Clostridium_sensu_stricto_1_15	2	21	136	458	0.0054	23-127	25-130	23-142
Lactobacillus_paracasei_18	1	1	166	-201	0.0285	NA	NA	NA
Fusicatenibacter_saccharivorans_20	1	NA	NA	NA	NA	pos 78-384	NA	pos 364-403
Blautia_25	1	33	411	-433	0.0317	NA	34-411	NA
Akkermansia_28	1	71	411	-338	0.0377	NA	NA	NA
Collinsella_24	1	1	356	-1172	0.013	1-334	1-248	1-354
Clostridium_sensu_stricto_1_23	1	1	12	-20	0.0054	1-7	NA	NA
Clostridium_sensu_stricto_1_23	2	25	92	172	0.0054	26-123	NA	NA
Clostridium_sensu_stricto_1_23	3	NA	NA	NA	NA	pos 364-369	NA	NA
Veillonella_29	1	97	197	-296	0.0054	26-289	104-180	52-208
Streptococcus_pyogenes_17	1	1	43	-17	0.0394	1-168	NA	NA
Clostridium_butyricum_26	1	1	385	690	0.013	5-241	1-411	1-363
Streptococcus_30	1	1	11	-16	0.0285	NA	NA	NA
Streptococcus_30	2	30	96	142	0.0098	NA	NA	NA
Peptostreptococcaceae_32	1	NA	NA	NA	NA	pos 51-135	NA	NA
Bacteroides_31	1	1	411	-606	0.008	1-384	1-220	27-242

Parabacteroides_37	1	NA	NA	NA	NA	NA	neg 83-411	NA
Bacteroides_33	1	1	411	-974	0.0054	1-384	1-411	1-403
Bifidobacterium_animalis_36	1	1	23	-51	0.0054	1-24	1-20	1-24
Bifidobacterium_animalis_36	2	47	97	89	0.0161	NA	46-117	NA
Clostridium_butyricum_35	1	1	14	-15	0.0098	1-5	NA	1-14
Clostridium_butyricum_35	2	40	140	135	0.0452	37-146	NA	87-205
Clostridium_paraputrificum_41	1	56	153	148	0.0117	51-369	75-113	NA
Clostridium_paraputrificum_41	2	353	376	31	0.0054	NA	363-374	NA
Lachnospiraceae_39	1	38	411	-411	0.0222	NA	56-411	174-403
Haemophilus_38	1	1	8	-10	0.0145	1-13	NA	NA
Haemophilus_38	2	44	86	70	0.0054	43-75	NA	NA
Lactobacillus_51	1	1	290	-126	0.013	26-289	1-262	1-182
Rothia_43	1	1	6	-6	0.0417	NA	1-17	*24-125
Rothia_43	2	27	125	189	0.0054	NA	59-102	NA
Lactobacillus_fermentum_49	1	1	120	-58	0.0285	NA	1-231	NA
Streptococcus_anginosus_subsp_whileyi_55	1	1	88	-98	0.0054	1-79	1-65	1-105
Akkermansia_64	1	1	306	-124	0.0145	34-384	NA	31-148
Bacteroides_58	1	8	411	-335	0.0054	81-384	8-340	110-403
Bacillus_cereus_68	1	1	141	21	0.0206	NA	NA	NA
Moryella_60	1	NA	NA	NA	NA	NA	neg 142-378	NA
Corynebacterium_47	1	1	144	57	0.0377	1-170	NA	NA
Bifidobacterium_63	1	1	307	-411	0.0098	1-361	1-260	1-162
Gardnerella_vaginalis_0288E_71	1	1	177	-40	0.0098	NA	1-150	NA
Roseburia_72	1	NA	NA	NA	NA	NA	neg 356-369	neg 243-364
Roseburia_72	2	371	411	284	0.0394	NA	376-411	371-403
Bacteroides_70	1	1	371	-464	0.0098	1-335	1-260	1-301
Lachnospiraceae_86	1	NA	NA	NA	NA	pos 203-384	NA	NA
Megamonas_46	1	NA	NA	NA	NA	NA	neg 164-411	pos 1-257
Lactococcus_lactis_78	1	NA	NA	NA	NA	pos 1-167	pos 74-310	NA
Peptostreptococcaceae_75	1	1	138	-71	0.0161	NA	1-96	52-155
Erysipelotrichaceae_74	1	2	411	-255	0.0098	80-384	78-411	67-293
Actinomyces_73	1	17	170	-73	0.046	43-197	NA	NA
Ruminococcaceae_90	1	NA	NA	NA	NA	NA	NA	neg 319-355
Ruminococcaceae_90	2	NA	NA	NA	NA	NA	NA	pos 383-403
Lachnospiraceae_103	1	NA	NA	NA	NA	pos 120-384	NA	NA
Veillonella_76	1	1	25	-34	0.008	1-51	*81-139	1-42
Tepidimonas_95	1	1	27	24	0.0054	1-15	NA	NA
Peptostreptococcus_98	1	1	183	-154	0.0098	1-166	1-155	1-140
Peptostreptococcus_98	2	364	411	36	0.0311	NA	396-411	NA
Clostridium_nexile_89	1	127	220	-610	0.008	NA	NA	NA

Clostridium_nexile_89	2	257	352	-749	0.0145	NA	NA	NA
Clostridium_nexile_89	3	366	411	266	0.0054	NA	NA	NA
Eubacterium_sp_CS1_Van_96	1	NA	NA	NA	NA	NA	NA	pos 1-279
Corynebacterium_striatum_77	1	105	411	135	0.0054	61-384	NA	NA
Stenotrophomonas_maltophilia_91	1	1	139	28	0.0195	1-185	NA	NA
Blautia_112	1	1	11	-3	0.0098	NA	NA	*223-403
Blautia_112	2	176	411	124	0.0275	NA	NA	NA
Enhydrobacter_119	1	1	42	20	0.0054	1-33	NA	NA
Clostridium_sensu_stricto_1_124	1	14	120	39	0.0357	1-171	NA	NA
Veillonella_87	1	1	37	-29	0.0054	1-35	1-5	1-121
Coprococcus_99	1	110	411	-237	0.023	NA	NA	193-403
Atopobium_138	1	1	411	-88	0.0054	97-206	95-208	95-209
Bacteroides_48	1	1	228	-98	0.008	46-315	1-95	1-189
Lachnospira_108	1	297	364	711	0.0054	NA	318-363	NA
Lachnospira_108	2	370	388	-28	0.0325	NA	367-374	NA
Parasutterella_88	1	151	411	-163	0.043	NA	NA	NA
Comamonadaceae_130	1	1	26	15	0.0054	1-27	NA	1-27
Nitriliruptor_126	1	1	26	14	0.0054	NA	NA	NA
Varibaculum_117	1	NA	NA	NA	NA	neg 64-214	NA	NA
Anaerococcus_105	1	NA	NA	NA	NA	NA	neg 17-51	pos 127-168
Bacteroides_94	1	NA	NA	NA	NA	NA	pos 1-127	NA
Lachnospiraceae_145	1	NA	NA	NA	NA	NA	neg 146-411	NA
Bilophila_wadsworthia_3_1_6_123	1	1	411	-489	0.0054	1-384	NA	1-403
Bifidobacteriaceae_122	1	1	134	-92	0.0336	NA	NA	1-143
Veillonella_115	1	1	13	-11	0.0098	1-13	*89-134	NA
Veillonella_115	2	95	136	40	0.0222	NA	NA	NA
Acinetobacter_143	1	1	28	19	0.0054	1-27	NA	1-34
Erysipelotrichaceae_148	1	1	7	-1	0.018	NA	NA	NA
Ruminococcus_sp_14531_180	1	NA	NA	NA	NA	pos 1-255	NA	NA
Subdoligranulum_136	1	NA	NA	NA	NA	NA	neg 40-150	NA
Acinetobacter_157	1	1	151	61	0.0098	1-178	NA	1-82
Aquabacterium_152	1	1	25	16	0.0054	NA	NA	NA
Bifidobacterium_146	1	1	193	-173	0.0054	1-148	1-172	1-173
Dorea_170	1	242	365	-581	0.0145	268-363	NA	NA
Dorea_170	2	373	411	135	0.0377	369-384	NA	NA
Coriobacteriaceae_bacterium_WAL_18889_151	1	1	373	-248	0.023	NA	1-411	NA
Phascolarctobacterium_165	1	1	153	-71	0.0054	NA	1-206	1-89
Phascolarctobacterium_165	2	NA	NA	NA	NA	NA	NA	neg 298-403
Sutterella_134	1	1	349	-197	0.023	1-381	NA	NA
Akkermansia_153	1	15	127	-35	0.0161	59-308	NA	NA

Burkholderia_156	1	1	27	9	0.0394	1-27	NA	NA
Escherichia_Shigella_154	1	1	25	-50	0.0054	1-29	1-16	1-39
Pseudomonas_fluorescens_144	1	1	28	10	0.0098	NA	NA	NA
Coprococcus_sp_DJF_B005_176	1	128	411	-102	0.0472	NA	NA	NA
Bifidobacterium_161	1	NA	NA	NA	NA	NA	NA	neg 88-234
Lachnospiraceae_188	1	82	411	-201	0.0262	NA	82-411	NA
Bifidobacteriaceae_193	1	NA	NA	NA	NA	NA	pos 30-74	NA
Eggerthella_178	1	1	190	-232	0.0098	1-160	1-150	4-189
Eggerthella_178	2	326	411	156	0.008	320-384	320-411	345-403
Bacteroides_194	1	99	411	-153	0.046	139-384	NA	NA
Gemella_120	1	1	38	-83	0.0054	1-32	1-40	1-32
Gemella_120	2	98	129	31	0.013	113-124	103-132	NA
Bifidobacterium_dentium_Bd1_179	1	1	8	-5	0.008	NA	*30-135	NA
Bifidobacterium_dentium_Bd1_179	2	39	131	86	0.0195	NA	NA	NA
Bacteroides_214	1	49	133	-39	0.0325	NA	13-122	NA
Bifidobacterium_233	1	1	206	-109	0.0117	1-189	NA	1-128
Streptococcus_192	1	1	17	-27	0.0054	1-18	1-18	1-9
Streptococcus_192	2	32	110	135	0.0098	49-62	105-121	34-103
Alistipes_215	1	7	358	-189	0.008	31-351	NA	NA
Lactobacillus_delbrueckii_subsp_bulgaricus_190	1	NA	NA	NA	NA	neg 155-384	NA	NA
Bifidobacterium_dentium_Bd1_198	1	NA	NA	NA	NA	NA	pos 54-134	NA
Bifidobacterium_animalis_202	1	1	25	-21	0.0054	1-51	NA	1-137
Turicibacter_220	1	307	368	-101	0.0417	NA	NA	NA
Turicibacter_220	2	397	411	30	0.0442	NA	NA	NA
Bacteroides_222	1	1	177	-91	0.023	NA	1-175	1-97
Akkermansia_196	1	16	126	-29	0.0161	71-350	NA	NA
Enterococcus_186	1	12	74	65	0.008	1-70	NA	1-123
Collinsella_tanakaei_163	1	1	184	-56	0.0206	32-132	NA	37-117
Actinobaculum_schaalii_FB123_CNA_2_239	1	22	156	-83	0.008	23-143	NA	21-137
Klebsiella_200	1	13	129	233	0.0054	15-132	16-130	12-113
Bifidobacterium_228	1	1	211	-145	0.0195	1-245	1-140	1-130
Bifidobacterium_223	1	1	146	-90	0.0394	NA	NA	1-109
Bacteroides_249	1	NA	NA	NA	NA	neg 277-384	NA	NA
Bifidobacterium_251	1	1	347	-288	0.023	1-239	1-270	1-336
Streptococcus_241	1	1	45	-17	0.013	NA	NA	NA
Ruminococcaceae_243	1	1	141	-75	0.013	NA	1-199	31-89
Bacteroides_210	1	1	411	-309	0.008	1-384	1-200	NA
Epulopiscium_208	1	NA	NA	NA	NA	NA	pos 83-411	NA
Paracoccus_266	1	1	25	7	0.0275	NA	NA	NA
Anaerostipes_269	1	NA	NA	NA	NA	NA	NA	neg 269-355
Anaerostipes_269	2	NA	NA	NA	NA	NA	NA	pos 366-403

Bacteroides_236	1	11	317	-129	0.0161	108-384	1-136	NA
Parabacteroides_distasonis_207	1	NA	NA	NA	NA	neg 1-292	NA	NA
Lactococcus_189	1	1	93	42	0.008	1-25	NA	1-130
Actinomyces_292	1	NA	NA	NA	NA	pos 149-271	NA	NA
Ruminococcaceae_287	1	209	411	-103	0.0347	187-384	NA	289-403
Collinsella_227	1	1	203	-68	0.013	NA	NA	1-229
Bacteroides_247	1	1	361	-220	0.013	1-312	1-216	NA
Alistipes_255	1	124	411	-133	0.0317	142-384	NA	NA
Collinsella_237	1	1	342	-793	0.0054	1-320	1-262	1-357
Barnesiella_276	1	NA	NA	NA	NA	neg 97-384	NA	NA
Veillonella_sp_DNF00869_201	1	1	21	-17	0.0054	1-22	1-19	1-15
Veillonella_sp_DNF00869_201	2	51	112	44	0.0054	106-120	NA	NA
Streptococcus_338	1	4	8	1	0.046	1-25	362-411	NA
Ruminococcaceae_306	1	NA	NA	NA	NA	NA	NA	neg 277-403
Roseburia_304	1	262	369	-352	0.0262	NA	NA	269-363
Roseburia_304	2	NA	NA	NA	NA	NA	NA	pos 373-403
Dermabacter_335	1	NA	NA	NA	NA	neg 1-163	NA	neg 31-143
ratAN060301C_268	1	1	296	-178	0.0117	1-364	1-197	NA
Streptococcus_303	1	1	162	77	0.0161	NA	1-144	1-109
Bacteroides_fragilis_CL03T00C08_291	1	1	329	-289	0.0098	13-384	6-267	24-183
Pasteurella_pneumotropica_301	1	NA	NA	NA	NA	neg 1-14	NA	NA
Ruminococcaceae_277	1	230	411	-132	0.0054	NA	215-411	222-403
Finegoldia_164	1	1	199	109	0.0347	1-229	NA	1-178
Acinetobacter_ursingii_ANC_3649_388	1	35	204	52	0.008	NA	NA	NA
Bifidobacteriaceae_290	1	1	28	-14	0.013	NA	NA	1-41
Lachnospiraceae_294	1	NA	NA	NA	NA	NA	neg 109-411	NA
Bacteroides_248	1	NA	NA	NA	NA	NA	neg 1-171	NA
Bacteroides_324	1	NA	NA	NA	NA	NA	neg 1-110	NA
Prevotella_361	1	95	411	-88	0.0145	NA	NA	NA
Oscillospiraceae_bacterium_VE202_24_316	1	NA	NA	NA	NA	NA	NA	neg 93-403
Dialister_365	1	1	397	-98	0.0336	77-384	75-411	NA
Gardnerella_332	1	1	305	-191	0.013	1-384	NA	1-189
Bifidobacteriaceae_320	1	98	219	-237	0.0098	94-175	104-198	51-227
Bifidobacterium_333	1	1	23	-41	0.0054	1-26	1-28	1-26
Streptococcus_gallolyticus_subsp_macedonicus_350	1	1	20	-6	0.0452	NA	NA	NA
Corynebacterium_328	1	1	7	4	0.0394	NA	NA	NA
Lachnospiraceae_326	1	NA	NA	NA	NA	NA	neg 106-411	NA
Escherichia_Shigella_331	1	1	18	-13	0.0054	1-9	NA	NA
Streptococcus_gallolyticus_subsp_macedonicus_386	1	1	184	-46	0.0145	1-173	NA	1-139
Ruminococcus_gnavus_CC55_001C_364	1	1	49	-22	0.0117	1-4	NA	1-130

Blautia_hydrogenotrophica_262	1	95	411	-108	0.0311	NA	NA	NA
Blautia_378	1	NA	NA	NA	NA	NA	neg 118-411	NA
Lactobacillus_371	1	1	106	-35	0.0054	1-140	1-95	1-90
Roseburia_383	1	246	370	-474	0.0206	NA	355-371	250-367
Roseburia_383	2	NA	NA	NA	NA	NA	pos 376-393	pos 376-403
Bifidobacterium_dentium_Bd1_395	1	22	73	31	0.0317	NA	NA	NA
Bifidobacteriaceae_344	1	1	15	-13	0.0054	1-10	1-6	1-9
Bifidobacteriaceae_344	2	34	71	36	0.0247	*120-134	NA	NA
Bifidobacteriaceae_344	3	124	143	-17	0.0206	NA	NA	NA
Bifidobacterium_368	1	1	225	-145	0.0054	2-211	1-143	1-216
Ruminococcus_gnavus_CC55_001C_366	1	1	220	-107	0.0275	NA	1-130	1-194
Veillonella_300	1	89	132	29	0.013	96-129	99-116	NA
Dialister_302	1	13	349	-79	0.0145	NA	NA	NA
Clostridium_sensu_stricto_1_341	1	1	3	-1	0.0054	NA	NA	*21-124
Clostridium_sensu_stricto_1_341	2	18	84	67	0.0054	NA	NA	NA
Lachnospiraceae_362	1	149	411	-97	0.0317	NA	NA	202-403
Fusobacterium_369	1	1	32	-12	0.0498	NA	NA	NA
Bifidobacterium_336	1	NA	NA	NA	NA	neg 45-216	NA	NA
Clostridium_sensu_stricto_1_355	1	1	7	-3	0.0098	*28-129	NA	NA
Clostridium_sensu_stricto_1_355	2	28	85	35	0.0098	NA	NA	NA
Leuconostoc_275	1	1	20	7	0.008	1-26	NA	*271-372
Leuconostoc_275	2	364	366	-1	0.0145	NA	NA	NA
Bifidobacterium_347	1	25	411	-173	0.0222	40-384	NA	NA
Coprobacter_443	1	1	252	-53	0.0371	49-210	NA	NA
Lachnospiraceae_419	1	NA	NA	NA	NA	pos 204-384	neg 312-366	neg 299-360
Lachnospiraceae_419	2	NA	NA	NA	NA	NA	NA	pos 366-400
Bacteroides_400	1	1	411	-205	0.0054	96-384	1-262	1-294
Streptococcus_gallolyticus_subsp_macedonicus_464	1	1	20	-12	0.0054	1-20	1-16	1-17
Leuconostoc_mesenteroides_282	1	1	22	12	0.0054	1-26	NA	1-204
Corynebacterium_propinquum_408	1	1	56	-27	0.0285	1-73	NA	NA
Lactobacillus_390	1	64	213	-85	0.0347	NA	NA	NA
Actinomyces_sp_oral_clone_DR002_406	1	NA	NA	NA	NA	pos 18-117	NA	NA
Subdoligranulum_479	1	NA	NA	NA	NA	NA	NA	neg 211-403
Plesiomonas_485	1	58	171	35	0.0247	39-200	NA	NA
Corynebacterium_freneyi_429	1	NA	NA	NA	NA	pos 1-143	NA	NA
Staphylococcus_427	1	1	46	-27	0.008	1-70	1-24	1-72
Clostridium_difficile_630_345	1	NA	NA	NA	NA	neg 1-11	NA	NA
Clostridium_difficile_630_359	1	NA	NA	NA	NA	NA	neg 25-120	NA
Clostridiales_bacterium_20_2a_461	1	27	395	-121	0.0117	NA	84-345	NA
Pseudobutyrivibrio_497	1	294	367	-178	0.0325	NA	NA	249-367

Bifidobacterium_breve_473	1	1	82	-40	0.0054	1-65	NA	1-166
Staphylococcaceae_452	1	1	42	15	0.0317	1-64	NA	NA
Bifidobacterium_466	1	NA	NA	NA	NA	NA	NA	pos 300-348
Streptococcus_521	1	1	175	-43	0.0301	1-178	1-125	NA
Escherichia_Shigella_469	1	19	226	-57	0.0417	1-168	NA	NA
Bacteroides_445	1	1	386	-190	0.008	1-384	NA	1-266
Clostridium_sensu_stricto_1_458	1	NA	NA	NA	NA	NA	NA	pos 117-161
Propionibacterium_434	1	3	67	-32	0.0357	NA	NA	8-91
Prevotella_buccae_ATCC_33574_586	2	366	374	4	0.0325	NA	NA	NA
Streptococcus_447	1	1	180	-61	0.023	NA	NA	NA
Bacteroides_493	1	1	338	-138	0.0117	38-247	6-185	1-267
Veillonella_502	1	NA	NA	NA	NA	neg 1-6	pos 1-228	NA
Barnesiella_516	1	70	411	-65	0.023	NA	NA	NA
Veillonella_454	1	NA	NA	NA	NA	NA	pos 74-344	NA
Blautia_484	1	90	411	-106	0.0336	NA	NA	93-403
Staphylococcus_507	1	1	18	-11	0.023	1-19	1-18	NA
Ruminococcaceae_532	1	NA	NA	NA	NA	NA	NA	neg 180-403
Bacteroides_482	1	41	411	-111	0.0206	37-111	NA	NA
Bacteroides_482	2	NA	NA	NA	NA	neg 165-384	NA	NA
Pseudobutyrivibrio_570	1	296	365	-261	0.008	NA	NA	246-367
Pseudobutyrivibrio_570	2	372	397	28	0.0311	NA	NA	376-403
Streptococcus_gallolyticus_subsp_macedonicus_554	1	19	106	-15	0.0325	NA	NA	3-201
Staphylococcus_489	1	1	23	-17	0.0054	NA	1-20	1-21
Actinomyces_europaeus_ACS_120_V_Col10b_541	1	36	212	-53	0.0317	NA	NA	NA
Bacilli_471	1	1	36	-33	0.0054	1-69	1-31	1-32
Campylobacter_646	1	19	113	40	0.0054	1-131	NA	NA
Bifidobacterium_animalis_581	1	NA	NA	NA	NA	NA	NA	neg 1-70
Bacteroides_603	1	47	282	-54	0.0377	125-384	NA	NA
Bacteroides_645	1	NA	NA	NA	NA	pos 1-118	NA	NA
Veillonella_555	1	34	63	12	0.039	NA	NA	NA
Anaerococcus_418	1	1	120	15	0.0357	NA	NA	NA
Bacillales_615	1	NA	NA	NA	NA	NA	neg 1-21	NA
Clostridium_sensu_stricto_1_110	1	81	271	60	0.0285	118-136	67-352	NA
Prevotella_melaninogenica_639	1	1	53	-15	0.0317	NA	NA	NA
Bacteroides_535	1	72	411	-100	0.013	89-384	NA	143-403
Peptococcus_664	1	NA	NA	NA	NA	NA	neg 52-135	NA
Ruminococcus_sp_CE2_596	1	102	411	-124	0.0206	107-384	NA	115-403
Corynebacteriaceae_504	1	38	131	-45	0.008	*1-13	NA	NA
Corynebacteriaceae_504	2	NA	NA	NA	NA	neg 39-122	NA	NA
Staphylococcus_565	1	1	21	-18	0.0054	1-23	1-18	1-22

Staphylococcus_565	2	NA	NA	NA	NA	NA	pos 50-65	NA
Methylobacterium_radiotolerans_437	1	1	273	-79	0.013	1-217	1-222	56-196
Lachnospiraceae_633	1	67	411	-77	0.0117	NA	NA	NA
Bifidobacterium_animalis_647	1	NA	NA	NA	NA	pos 9-264	NA	NA
Clostridium_sensu_stricto_1_648	1	42	154	37	0.0054	NA	45-144	NA
Bacteroides_658	1	70	366	-77	0.0206	64-384	NA	NA
Blautia_679	1	NA	NA	NA	NA	NA	neg 110-411	NA
Dialister_sp_oral_taxon_502_636	1	150	411	42	0.0117	NA	NA	NA
Bacteroides_635	1	28	299	-41	0.0275	NA	1-238	NA
Bacteroides_663	1	121	411	-52	0.018	NA	NA	227-403
Lachnospiraceae_652	1	124	137	-5	0.018	NA	NA	115-403
Lachnospiraceae_652	2	194	365	-467	0.0098	NA	NA	NA
Lachnospiraceae_652	3	373	411	77	0.0145	NA	NA	NA
Bifidobacterium_breve_634	1	NA	NA	NA	NA	NA	NA	pos 122-403
Akkermansia_671	1	49	215	-20	0.0394	NA	NA	NA
Lachnospiraceae_660	1	NA	NA	NA	NA	NA	neg 122-411	NA
Bacteroides_657	1	1	411	-120	0.008	1-314	1-310	1-274
Enterobacteriaceae_672	1	43	108	18	0.013	NA	50-175	NA
Clostridium_sensu_stricto_1_673	1	77	239	36	0.0195	42-242	NA	NA

Differential abundance testing by smoothing spline analysis of variance (SS-ANOVA) was executed to test in which specific intervals significant differences in OTUs existed between the groups, adjusted for mode of delivery. The OTUs are arranged in descending order based on their relative abundance in this dataset. A positive Area value indicates that the abundance of a specific OTU is higher in the antibiotic treated group, while a negative area value indicates that the abundance of that OTU is higher in the controls. To correct for multiple testing, the Benjamini-Hochberg method was applied, and the adjusted p-values (p.adj) are shown. The last three columns show the results of the comparisons between each regimen (ac = amoxicillin + cefotaxime, cg = co-amoxiclav + gentamicin, pg = penicillin + gentamicin) and controls, with the values representing interval in days where a statistically significant difference in abundance exists. NA indicates that for this comparison a specific OTU was not differentially abundant. An asterisk stands for an opposite direction of the effect compared to the value shown under Area. If the value for the antibiotic treated versus controls comparison is NA, but a statistically significant differential interval is found for an antibiotic regimen versus controls comparison, pos indicates that the abundance of a specific OTU is higher in the antibiotic regimen group, while neg indicates the abundance of that OTU is higher in the controls. Int.no = interval number. In some cases, multiple OTUs of individual bacterial species were identified, so OTUs are referred to by their taxonomical annotations and a rank number (shown in parentheses), which is based on the abundance of each given OTU in the overall dataset.

Supplementary Table 5: Correlation between top five most abundant 16S rRNA OTUs and MGS species

16S rRNA OTUs	MGS species	Pearson's r	p.adj
Bifidobacterium (1)	*Bifidobacterium adolescentis*<br>*Bifidobacterium breve*<br>*Bifidobacterium longum*	0.95	<0.001
Escherichia coli (2)	*Escherichia coli*	0.89	<0.001
Staphylococcus epidermidis (3)	*Staphylococcus epidermidis*	0.92	<0.001
Klebsiella (4)	*Klebsiella oxytoca*<br>*Klebsiella pneumoniae*	0.60	<0.001
Enterococcus faecium (5)	*Enterococcus faecium*	0.80	<0.001

The top five most abundant OTUs of the 16S rRNA dataset were correlated with the corresponding metagenomic shotgun sequencing (MGS) sequencing species using two-sided Pearson correlations. An adjusted p-value (p.adj, calculated using the Benjamini-Hochberg method) of <0.05 stands for a significant correlation (not a significant difference). Source data are provided as a Source Data file.

Supplementary Table 6: FitTimeSeries results of the significantly differentially abundant taxa between the amoxicillin + cefotaxime and co-amoxiclav + gentamicin regimens

OTU	Int.no	Interval start	Interval end	Area	p.adj
Escherichia_coli_2	1	3	54	175	0.0228
Klebsiella_4	1	3	17	33	0.0426
Peptostreptococcaceae_32	1	52	151	-198	0.0466
Lactobacillus_40	1	3	19	-11	0.0228
Corynebacterium_47	1	3	20	-20	0.0466
Tepidimonas_95	1	3	26	-32	0.0426
Clostridium_sensu_stricto_1_124	1	3	111	-85	0.0426
Comamonadaceae_130	1	3	25	-20	0.0492
Acinetobacter_143	1	3	25	-18	0.0466
Ruminococcus_sp_14531_180	1	19	138	-54	0.0466
Lachnospiraceae_175	1	254	368	-944	0.0466
Lachnospiraceae_175	2	375	411	235	0.0228
Bifidobacteriaceae_193	1	23	69	62	0.0426
Bifidobacteriaceae_193	2	374	410	105	0.0492
Bacteroides_236	1	252	411	125	0.0466
Parabacteroides_distasonis_207	1	3	178	118	0.0228
Finegoldia_164	1	3	29	-27	0.0466
Anaerococcus_tetradius_329	1	3	47	-23	0.0466
Bacteroides_248	1	3	127	-48	0.0228
Leuconostoc_275	1	3	30	-21	0.032
Bacteroides_380	1	3	66	-19	0.0466
Actinomyces_sp_oral_clone_DR002_406	1	15	70	-59	0.0228
Lachnospiraceae_415	1	376	403	-127	0.0426
Dorea_531	1	164	411	-149	0.032
Veillonella_454	1	309	411	89	0.0228
Bacteroides_482	1	80	411	130	0.032

Differential abundance testing by smoothing spline analysis of variance (SS-ANOVA) was executed to test in which specific intervals significant differences in OTUs existed between the groups, adjusted for prepartum maternal antibiotics. The OTUs are arranged in descending order based on their relative abundance in this dataset. A positive Area value indicates that the abundance of a specific OTU is higher in the co-amoxiclav + gentamicin regimen, while a negative area value indicates that the abundance of that OTU is higher in the amoxicillin + cefotaxime regimen. To correct for multiple testing, the Benjamini-Hochberg method was applied, and the adjusted p-values (p.adj) are shown. Int.no = interval number. In some cases, multiple OTUs of individual bacterial species were identified, so OTUs are referred to by their taxonomical annotations and a rank number (shown in parentheses), which is based on the abundance of each given OTU in the overall dataset.

Supplementary Table 7: FitTimeSeries results of the significantly differentially abundant taxa between the amoxicillin + cefotaxime and penicillin + gentamicin regimens

OTU	Int.no	Interval start	Interval end	Area	p.adj
Klebsiella_4	1	2	16	35	0.0457
Klebsiella_4	2	68	121	-126	0.0426
Ruminococcus_gnavus_CC55_001C_10	1	39	273	-495	0.047
Prevotella_27	1	2	185	147	0.016
Peptostreptococcaceae_32	2	90	195	-243	0.0457
Subdoligranulum_45	1	302	403	-185	0.0457
Lactobacillus_42	1	89	160	87	0.0457
Peptostreptococcaceae_50	1	121	403	-357	0.0457
Bifidobacterium_63	1	14	320	283	0.0457
Blautia_79	1	280	367	-549	0.0426
Ruminococcaceae_90	1	269	366	-714	0.0457
Ruminococcaceae_90	2	376	403	136	0.047
Eubacterium_sp_CS1_Van_96	1	2	360	231	0.016
Bilophila_wadsworthia_3_1_6_123	1	2	166	69	0.0492
Bifidobacteriaceae_122	1	22	43	-21	0.0457
Lachnospiraceae_162	1	106	403	-179	0.0457
Eubacterium_desmolans_125	1	253	368	-938	0.032
Sutterella_134	1	2	16	9	0.0457
Roseburia_185	1	372	403	88	0.0457
Escherichia_Shigella_154	1	2	17	19	0.0457
Gemella_120	1	201	403	-165	0.047
Lactobacillales_142	1	2	52	-28	0.0457
Lachnospiraceae_253	1	281	361	-286	0.032
Lachnospiraceae_253	2	370	403	100	0.0457
Bacteroides_210	1	124	403	206	0.016
Sutterella_197	1	71	210	-112	0.0457
Anaerostipes_269	1	273	368	-734	0.0284
Anaerostipes_269	2	376	403	98	0.016
Parabacteroides_distasonis_207	1	2	166	87	0.0457
Actinomyces_292	1	13	189	-81	0.047
Ruminococcaceae_306	1	134	403	-221	0.0457
ratAN060301C_268	1	44	231	84	0.0476
Bifidobacterium_animalis_293	1	52	403	-214	0.0457
Gardnerella_332	1	326	403	66	0.047
Bifidobacterium_336	1	43	289	107	0.047
Leuconostoc_275	1	2	25	-15	0.0393
Bacteroides_400	1	2	21	-6	0.0457

Subdoligranulum_479	1	147	403	-129	0.0457
Clostridium_difficile_630_345	1	2	86	65	0.016
Clostridium_difficile_630_359	1	2	39	23	0.016
Pseudobutyrivibrio_497	1	259	370	-456	0.047
Streptococcus_521	1	36	111	513	0.016
Streptococcus_521	2	123	124	-1	0.016
Veillonella_465	1	125	403	130	0.0476
Lachnospiraceae_415	2	366	403	-149	0.0457
Peptostreptococcaceae_396	1	2	205	93	0.0457
Veillonella_454	1	107	129	-12	0.0457
Veillonella_454	2	345	403	53	0.032
Lachnospiraceae_512	1	106	403	-200	0.0457
Fusicatenibacter_saccharivorans_606	1	290	370	-181	0.0457
Fusicatenibacter_saccharivorans_606	2	394	403	16	0.0457
Bifidobacterium_animalis_591	1	87	303	-60	0.0457

Differential abundance testing by smoothing spline analysis of variance (SS-ANOVA) was executed to test in which specific intervals significant differences in OTUs existed between the groups, adjusted for prepartum maternal antibiotics. The OTUs are arranged in descending order based on their relative abundance in this dataset. A positive Area value indicates that the abundance of a specific OTU is higher in the penicillin + gentamicin regimen, while a negative area value indicates that the abundance of that OTU is higher in the amoxicillin + cefotaxime regimen. To correct for multiple testing, the Benjamini-Hochberg method was applied, and the adjusted p-values (p.adj) are shown. Int.no = interval number. In some cases, multiple OTUs of individual bacterial species were identified, so OTUs are referred to by their taxonomical annotations and a rank number (shown in parentheses), which is based on the abundance of each given OTU in the overall dataset.

Supplementary Table 8: FitTimeSeries results of the significantly differentially abundant taxa between the co-amoxiclav + gentamicin and penicillin + gentamicin regimens

OTU	Int.no	Interval start	Interval end	Area	p.adj
Escherichia_coli_2	1	2	43	-74	0.0389
Megamonas_46	1	26	411	291	0.0214
Clostridium_sensu_stricto_1_65	1	31	282	316	0.0267
Lachnospiraceae_175	1	344	357	-66	0.0267
Lachnospiraceae_175	3	373	411	-241	0.0214
Lactobacillus_delbrueckii_subsp_bulgaricus_190	1	255	411	155	0.0321
Oscillospiraceae_bacterium_VE202_24_316	1	81	411	-318	0.0214
Streptococcus_gallolyticus_subsp_macedonicus_350	1	102	177	-53	0.0493
Lachnospiraceae_362	1	124	411	-167	0.0499
Ruminococcus_sp_CE2_417	1	109	411	299	0.0321
Subdoligranulum_479	1	149	411	-193	0.0267
Pseudobutyrivibrio_497	1	344	358	-37	0.0214
Pseudobutyrivibrio_497	2	366	374	10	0.0214
Bifidobacterium_breve_473	1	2	189	-82	0.0445
Clostridium_sensu_stricto_1_458	1	102	185	31	0.0499

Differential abundance testing by smoothing spline analysis of variance (SS-ANOVA) was executed to test in which specific intervals significant differences in OTUs existed between the groups, adjusted for prepartum maternal antibiotics. The OTUs are arranged in descending order based on their relative abundance in this dataset. A positive Area value indicates that the abundance of a specific OTU is higher in the penicillin + gentamicin regimen, while a negative area value indicates that the abundance of that OTU is higher in the co-amoxiclav + gentamicin regimen. To correct for multiple testing, the Benjamini-Hochberg method was applied, and the adjusted p-values (p.adj) are shown. Int.no = interval number. In some cases, multiple OTUs of individual bacterial species were identified, so OTUs are referred to by their taxonomical annotations and a rank number (shown in parentheses), which is based on the abundance of each given OTU in the overall dataset.

Supplementary Table 9: FitTimeSeries results of the significantly differentially abundant taxa between the amoxicillin + cefotaxime regimen and controls without adjusting for breastfeeding (detailed subset of Supplementary Table 3)

OTU	Int.no	Interval start	Interval end	Area	p.adj
Bifidobacterium_1	1	1	43	-322.223	0.006327
Escherichia_coli_2	1	1	142	-383.918	0.006327
Staphylococcus_epidermidis_3	1	12	175	-190.848	0.024883
Klebsiella_4	1	16	127	412.0667	0.008973
Enterococcus_faecium_5	1	1	117	330.4863	0.006327
Streptococcus_salivarius_subsp_thermophilus_6	1	1	27	-96.3985	0.006327
ratAN060301C_7	1	1	343	-797.615	0.01677
Veillonella_9	1	119	127	19.92105	0.006327
Veillonella_9	2	4	17	-80.0658	0.006327
Ruminococcus_gnavus_CC55_001C_10	1	1	64	-108.761	0.011216
Streptococcus_11	1	1	384	676.7696	0.01316
Bifidobacterium_12	1	1	18	-47.0536	0.006327
Bifidobacterium_bifidum_NCIMB_41171_13	1	1	213	-630.168	0.006327
Clostridium_sensu_stricto_1_15	1	23	127	397.3748	0.011216
Clostridium_sensu_stricto_1_15	2	1	10	-22.6993	0.006327
Streptococcus_pyogenes_17	1	1	168	-93.9667	0.008973
Fusicatenibacter_saccharivorans_20	1	78	384	727.0451	0.014688
Clostridium_sensu_stricto_1_23	1	26	123	250.3941	0.006327
Clostridium_sensu_stricto_1_23	2	364	369	7.9566	0.027859
Clostridium_sensu_stricto_1_23	3	1	7	-11.5374	0.006327
Collinsella_24	1	1	334	-1214.79	0.008973
Clostridium_butyricum_26	1	5	241	626.4973	0.01677
Veillonella_29	1	26	289	-382.257	0.041402
Bacteroides_31	1	1	384	-820.812	0.008973
Peptostreptococcaceae_32	1	51	135	169.9822	0.01592
Bacteroides_33	1	1	384	-955.142	0.01316
Clostridium_butyricum_35	1	37	146	215.8418	0.011216
Clostridium_butyricum_35	2	1	5	-4.74036	0.029685
Bifidobacterium_animalis_36	1	1	24	-47.4711	0.011216
Haemophilus_38	1	43	75	78.78951	0.006327
Haemophilus_38	2	1	13	-26.2015	0.006327
Clostridium_paraputrificum_41	1	51	369	781.9406	0.01677
Corynebacterium_47	1	1	170	110.201	0.01677
Bacteroides_48	1	46	315	-129.396	0.011216
Lactobacillus_51	1	26	289	-124.669	0.038501
Streptococcus_anginosus_subsp_whileyi_55	1	1	79	-98.3452	0.008973
Bacteroides_58	1	81	384	-257.989	0.02402
Bifidobacterium_63	1	1	361	-626.805	0.006327

Akkermansia_64	1	34	384	-201.447	0.024883
Bacteroides_70	1	1	335	-436.998	0.011216
Actinomyces_73	1	43	197	-124.623	0.038501
Erysipelotrichaceae_74	1	80	384	-210.526	0.032659
Veillonella_76	1	1	51	-73.1769	0.01592
Corynebacterium_striatum_77	1	61	384	218.3096	0.006327
Lactococcus_lactis_78	1	1	167	122.3168	0.014688
Lachnospiraceae_86	1	203	384	316.7736	0.028472
Veillonella_87	1	1	35	-32.5948	0.011216
Stenotrophomonas_maltophilia_91	1	1	185	63.93591	0.014688
Tepidimonas_95	1	1	15	33.08484	0.006327
Peptostreptococcus_98	1	1	166	-143.485	0.020755
Lachnospiraceae_103	1	120	384	236.7884	0.024883
Clostridium_sensu_stricto_1_110	1	118	136	8.877937	0.024883
Veillonella_115	1	1	13	-11.0155	0.028472
Varibaculum_117	1	64	214	-138.246	0.044289
Enhydrobacter_119	1	1	33	32.62138	0.006327
Gemella_120	1	113	124	11.52539	0.01592
Gemella_120	2	1	32	-74.6978	0.006327
Bilophila_wadsworthia_3_1_6_123	1	1	384	-629.623	0.008973
Clostridium_sensu_stricto_1_124	1	1	171	122.3527	0.022848
Comamonadaceae_130	1	1	27	27.86178	0.006327
Sutterella_134	1	1	381	-305.212	0.01592
Atopobium_138	1	97	206	-22.3409	0.01677
Acinetobacter_143	1	1	27	28.25223	0.006327
Bifidobacterium_146	1	1	148	-147.088	0.014688
Akkermansia_153	1	59	308	-99.4841	0.040842
Escherichia_Shigella_154	1	1	29	-77.5914	0.006327
Burkholderia_156	1	1	27	19.02483	0.006327
Acinetobacter_157	1	1	178	107.8788	0.008973
Collinsella_tanakaei_163	1	32	132	-35.9938	0.040842
Finegoldia_164	1	1	229	189.322	0.006327
Dorea_170	1	369	384	49.99015	0.006327
Dorea_170	2	268	363	-832.234	0.006327
Eggerthella_178	1	320	384	112.5965	0.014688
Eggerthella_178	2	1	160	-185.537	0.01316
Ruminococcus_sp_14531_180	1	1	255	100.3386	0.032659
Enterococcus_186	1	1	70	56.5941	0.037279
Lactococcus_189	1	1	25	31.82054	0.006327
Lactobacillus_delbrueckii_subsp_bulgaricus_190	1	155	384	-153.944	0.044289
Streptococcus_192	1	49	62	21.98578	0.014688
Streptococcus_192	2	1	18	-35.0849	0.006327
Bacteroides_194	1	139	384	-193.755	0.024883

Akkermansia_196	1	71	350	-107.308	0.037279
Klebsiella_200	1	15	132	287.9528	0.011216
Veillonella_sp_DNF00869_201	1	106	120	9.474116	0.006327
Veillonella_sp_DNF00869_201	2	1	22	-21.6387	0.008973
Bifidobacterium_animalis_202	1	1	51	-28.2932	0.043545
Parabacteroides_distasonis_207	1	1	292	-146.744	0.008973
Bacteroides_210	1	1	384	-435.607	0.008973
Alistipes_215	1	31	351	-208.304	0.01592
Bifidobacterium_228	1	1	245	-190.287	0.024883
Bifidobacterium_233	1	1	189	-123.484	0.011216
Bacteroides_236	1	108	384	-205.636	0.008973
Collinsella_237	1	1	320	-792	0.014688
Actinobaculum_schaalii_FB123_CNA_2_239	1	23	143	-114.071	0.006327
Bacteroides_247	1	1	312	-227.701	0.008973
Bacteroides_249	1	277	384	-69.7358	0.02402
Bifidobacterium_251	1	1	239	-219.754	0.020755
Alistipes_255	1	142	384	-135.448	0.04715
ratAN060301C_268	1	1	364	-319.661	0.006327
Leuconostoc_275	1	1	26	21.07769	0.006327
Barnesiella_276	1	97	384	-133.937	0.028472
Leuconostoc_mesenteroides_282	1	1	26	23.90294	0.006327
Ruminococcaceae_287	1	187	384	-124.146	0.027859
Bacteroides_fragilis_CL03T00C08_291	1	13	384	-280.553	0.01592
Actinomyces_292	1	149	271	49.67905	0.041402
Veillonella_300	1	96	129	25.69363	0.028472
Pasteurella_pneumotropica_301	1	1	14	-10.2457	0.014688
Bifidobacteriaceae_320	1	94	175	-93.106	0.01677
Escherichia_Shigella_331	1	1	9	-7.27705	0.006327
Gardnerella_332	1	1	384	-306.135	0.01592
Bifidobacterium_333	1	1	26	-41.4159	0.006327
Dermabacter_335	1	1	163	-59.2534	0.040842
Bifidobacterium_336	1	45	216	-54.2568	0.028472
Streptococcus_338	1	1	25	10.48079	0.02402
Bifidobacteriaceae_344	1	1	10	-7.41766	0.008973
Bifidobacteriaceae_344	2	120	134	-12.1892	0.01592
Clostridium_difficile_630_345	1	1	11	-3.72434	0.01316
Bifidobacterium_347	1	40	384	-226.351	0.01677
Clostridium_sensu_stricto_1_355	1	28	129	77.54359	0.008973
Ruminococcus_gnavus_CC55_001C_364	1	1	4	-1.75659	0.039967
Dialister_365	1	77	384	-103.768	0.008973
Bifidobacterium_368	1	2	211	-161.21	0.006327
Lactobacillus_371	1	1	140	-42.2732	0.01677
Streptococcus_gallolyticus_subsp_macedonicus_386	1	1	173	-54.0763	0.01316

Bacteroides_400	1	96	384	-163.619	0.011216
Actinomyces_sp_oral_clone_DR002_406	1	18	117	116.169	0.028472
Corynebacterium_propinquum_408	1	1	73	-50.679	0.042771
Lachnospiraceae_419	1	204	384	162.7779	0.044289
Staphylococcus_427	1	1	70	-32.7776	0.027859
Corynebacterium_freneyi_429	1	1	143	34.69555	0.029685
Methylobacterium_radiotolerans_437	1	1	217	-62.3588	0.041402
Coprobacter_443	1	49	210	-41.0252	0.026732
Bacteroides_445	1	1	384	-242.879	0.011216
Staphylococcaceae_452	1	1	64	35.06888	0.029685
Streptococcus_gallolyticus_subsp_macedonicus_464	1	1	20	-13.4726	0.01316
Escherichia_Shigella_469	1	1	168	-59.9257	0.041402
Bacilli_471	1	1	69	-46.4507	0.006327
Bifidobacterium_breve_473	1	1	65	-47.0128	0.006327
Bacteroides_482	1	37	111	-19.3429	0.01316
Bacteroides_482	2	165	384	-107.393	0.014688
Plesiomonas_485	1	39	200	91.10655	0.01316
Bacteroides_493	1	38	247	-80.1326	0.02402
Veillonella_502	1	1	6	-2.75991	0.01316
Corynebacteriaceae_504	1	1	13	7.0087	0.008973
Corynebacteriaceae_504	2	39	122	-64.5451	0.01677
Staphylococcus_507	1	1	19	-12.4752	0.043545
Streptococcus_521	1	1	178	-59.45	0.02402
Bacteroides_535	1	89	384	-85.344	0.018981
Staphylococcus_565	1	1	23	-23.2521	0.006327
Ruminococcus_sp_CE2_596	1	107	384	-132.235	0.020755
Bacteroides_603	1	125	384	-92.2238	0.01592
Bacteroides_645	1	1	118	28.56168	0.037279
Campylobacter_646	1	1	131	31.06805	0.043545
Bifidobacterium_animalis_647	1	9	264	52.5902	0.01677
Bacteroides_657	1	1	314	-101.568	0.006327
Bacteroides_658	1	64	384	-105.136	0.032659
Clostridium_sensu_stricto_1_673	1	42	242	49.04653	0.027859

Differential abundance testing by smoothing spline analysis of variance (SS-ANOVA) was executed to test in which specific intervals significant differences in OTUs existed between the groups, adjusted for mode of delivery. The OTUs are arranged in descending order based on their relative abundance in this dataset. A positive Area value indicates that the abundance of a specific OTU is higher in the amoxicillin + cefotaxime regimen, while a negative area value indicates that the abundance of that OTU is higher in the controls. To correct for multiple testing, the Benjamini-Hochberg method was applied, and the adjusted p-values (p.adj) are shown. Int.no = interval number. In some cases, multiple OTUs of individual bacterial species were identified, so OTUs are referred to by their taxonomical annotations and a rank number (shown in parentheses), which is based on the abundance of each given OTU in the overall dataset.

Supplementary Table 10: FitTimeSeries results of the significantly differentially abundant taxa between the amoxicillin + cefotaxime regien and controls while adjusting for breastfeeding

OTU	Int.no	Interval start	Interval end	Area	p.adj
Bifidobacterium_1	1	1	43	-324.479	0.005386
Escherichia_coli_2	1	1	48	-158.265	0.005386
Klebsiella_4	1	16	125	389.7467	0.010182
Enterococcus_faecium_5	1	1	94	251.3869	0.005386
Streptococcus_salivarius_subsp_thermophilus_6	1	1	28	-101.612	0.005386
ratAN060301C_7	1	1	329	-730.462	0.02082
Veillonella_9	1	120	126	14.81322	0.005386
Veillonella_9	2	3	17	-79.5033	0.005386
Ruminococcus_gnavus_CC55_001C_10	1	1	61	-103.917	0.010182
Streptococcus_11	1	1	384	727.6015	0.018018
Bifidobacterium_12	1	1	18	-47.5474	0.005386
Bifidobacterium_bifidum_NCIMB_41171_13	1	1	207	-600.948	0.008123
Clostridium_sensu_stricto_1_15	1	23	127	388.411	0.008123
Clostridium_sensu_stricto_1_15	2	1	10	-22.6441	0.011798
Streptococcus_pyogenes_17	1	1	168	-94.5789	0.008123
Fusicatenibacter_saccharivorans_20	1	84	384	698.8715	0.028154
Clostridium_sensu_stricto_1_23	1	29	81	127.6938	0.005386
Clostridium_sensu_stricto_1_23	2	1	8	-13.0972	0.008123
Collinsella_24	1	1	342	-1325.47	0.008123
Clostridium_butyricum_26	1	4	241	590.5183	0.010182
Akkermansia_28	1	101	384	-386.684	0.046267
Bacteroides_31	1	1	384	-829.847	0.011798
Bacteroides_33	1	1	384	-906.642	0.008123
Clostridium_butyricum_35	1	43	141	172.7327	0.005386
Clostridium_butyricum_35	2	1	6	-5.78975	0.019389
Bifidobacterium_animalis_36	1	1	25	-50.8092	0.005386
Haemophilus_38	1	30	132	278.5522	0.005386
Haemophilus_38	2	1	12	-24.1688	0.008123
Lactobacillus_40	1	35	62	-25.5163	0.015169
Clostridium_paraputrificum_41	1	57	363	687.7779	0.015169
Corynebacterium_47	1	1	181	123.3441	0.011798
Bacteroides_48	1	36	334	-151.544	0.005386
Lactobacillus_51	1	1	329	-175.345	0.034918
Streptococcus_anginosus_subsp_whileyi_55	1	1	68	-82.4844	0.013764
Bacteroides_58	1	74	384	-272.878	0.015169
Bifidobacterium_63	1	1	343	-564.998	0.008123
Akkermansia_64	1	15	384	-229.221	0.013764
Bacteroides_70	1	1	338	-444.768	0.010182

Roseburia_72	1	368	384	48.52357	0.046267
Actinomyces_73	1	49	184	-102.652	0.046267
Erysipelotrichaceae_74	1	81	384	-210.443	0.044351
Peptostreptococcaceae_75	1	368	384	65.41373	0.005386
Peptostreptococcaceae_75	2	126	134	-16.3938	0.005386
Peptostreptococcaceae_75	3	316	361	-465.417	0.005386
Veillonella_76	1	1	54	-77.2432	0.010182
Corynebacterium_striatum_77	1	64	384	214.516	0.005386
Lactococcus_lactis_78	1	1	16	16.38635	0.034918
Lachnospiraceae_86	1	214	384	301.315	0.032083
Veillonella_87	1	1	39	-36.8104	0.005386
Stenotrophomonas_maltophilia_91	1	1	196	71.06601	0.005386
Tepidimonas_95	1	1	27	48.33798	0.005386
Tepidimonas_95	2	51	76	-25.0645	0.005386
Peptostreptococcus_98	1	1	177	-159.217	0.02082
Coprococcus_99	1	140	384	-187.329	0.039201
Lachnospiraceae_103	1	122	384	234.6748	0.017003
Clostridium_sensu_stricto_1_110	1	119	136	8.357675	0.024171
Veillonella_115	1	1	16	-13.6335	0.018018
Enhydrobacter_119	1	1	33	33.19319	0.005386
Gemella_120	1	110	127	19.03825	0.005386
Gemella_120	2	1	32	-73.8688	0.005386
Bifidobacteriaceae_122	1	1	134	-119.583	0.029147
Bilophila_wadsworthia_3_1_6_123	1	1	384	-598.592	0.005386
Clostridium_sensu_stricto_1_124	1	1	163	114.8586	0.010182
Comamonadaceae_130	1	1	27	28.35392	0.005386
Sutterella_134	1	1	381	-305.891	0.015169
Atopobium_138	1	126	174	-9.43428	0.03052
Acinetobacter_143	1	1	27	28.58536	0.005386
Bifidobacterium_146	1	1	166	-176.161	0.010182
Akkermansia_153	1	51	324	-114.827	0.025766
Escherichia_Shigella_154	1	1	29	-76.9747	0.005386
Burkholderia_156	1	1	27	19.42555	0.005386
Acinetobacter_157	1	1	181	111.947	0.008123
Collinsella_tanakaei_163	1	30	138	-40.375	0.018018
Finegoldia_164	1	1	134	117.3084	0.005386
Dorea_170	1	369	384	49.74354	0.013764
Dorea_170	2	267	363	-847.459	0.013764
Eggerthella_178	1	319	384	114.4042	0.022711
Eggerthella_178	2	1	157	-179.476	0.015169
Ruminococcus_sp_14531_180	1	16	212	67.83765	0.029147
Enterococcus_186	1	1	44	31.95333	0.037737
Lactococcus_189	1	1	23	30.36847	0.005386

Lactobacillus_delbrueckii_subsp_bulgaricus_190	1	152	384	-158.129	0.044351
Streptococcus_192	1	1	20	-37.9859	0.005386
Bacteroides_194	1	133	384	-203.277	0.013764
Akkermansia_196	1	62	371	-125.98	0.015169
Klebsiella_200	1	15	131	272.8402	0.005386
Veillonella_sp_DNF00869_201	1	78	126	39.56797	0.005386
Veillonella_sp_DNF00869_201	2	1	21	-20.473	0.005386
Bifidobacterium_animalis_202	1	1	74	-39.9736	0.033939
Parabacteroides_distasonis_207	1	1	245	-105.104	0.011798
Bacteroides_210	1	10	384	-416.578	0.010182
Alistipes_215	1	37	342	-190.377	0.018018
Bifidobacterium_223	1	1	177	-152.024	0.018018
Collinsella_227	1	20	162	-52.3616	0.03315
Bifidobacterium_228	1	1	279	-241.208	0.011798
Bifidobacterium_233	1	1	175	-109.88	0.015169
Bacteroides_236	1	117	384	-197.459	0.010182
Collinsella_237	1	1	334	-871.444	0.011798
Actinobaculum_schaalii_FB123_CNA_2_239	1	25	139	-99.1253	0.008123
Streptococcus_241	1	1	74	-33.4764	0.008123
Bacteroides_247	1	1	286	-192.724	0.008123
Bacteroides_249	1	262	384	-79.917	0.018018
Bifidobacterium_251	1	1	264	-263.264	0.025766
Alistipes_255	1	132	384	-145.27	0.039201
ratAN060301C_268	1	1	328	-257.643	0.005386
Leuconostoc_275	1	1	24	19.91756	0.005386
Leuconostoc_275	2	118	130	-6.31147	0.005386
Barnesiella_276	1	96	384	-136.028	0.019389
Ruminococcaceae_277	1	136	384	-123.329	0.039201
Leuconostoc_mesenteroides_282	1	1	25	23.34859	0.005386
Lactobacillales_284	1	38	301	-279.476	0.02082
Ruminococcaceae_287	1	197	384	-119.482	0.03315
Bifidobacteriaceae_290	1	1	17	-9.20944	0.026888
Bacteroides_fragilis_CL03T00C08_291	1	23	382	-264.105	0.029147
Actinomyces_292	1	169	241	28.24573	0.034918
Pasteurella_pneumotropica_301	1	1	13	-9.24825	0.013764
Bifidobacteriaceae_320	1	83	190	-141.005	0.011798
Escherichia_Shigella_331	1	1	9	-7.23429	0.008123
Gardnerella_332	1	1	384	-326.213	0.011798
Bifidobacterium_333	1	1	26	-41.6445	0.005386
Dermabacter_335	1	1	171	-64.4005	0.039201
Bifidobacterium_336	1	49	212	-51.6362	0.026888
Streptococcus_338	1	1	25	10.74031	0.019389
Clostridium_sensu_stricto_1_341	1	1	118	45.1547	0.033939

Bifidobacteriaceae_344	1	1	10	-7.4067	0.005386
Bifidobacteriaceae_344	2	117	137	-18.5781	0.017003
Clostridium_difficile_630_345	1	1	78	-28.104	0.018018
Bifidobacterium_347	1	42	384	-225.723	0.018018
Clostridium_sensu_stricto_1_355	1	30	125	68.66705	0.005386
Dialister_365	1	63	384	-114.24	0.010182
Bifidobacterium_368	1	3	221	-184.418	0.005386
Lactobacillus_371	1	1	95	-27.6677	0.033939
Streptococcus_gallolyticus_subsp_macedonicus_386	1	1	181	-58.547	0.011798
Lactobacillus_390	1	79	220	-117.975	0.037737
Peptostreptococcaceae_396	1	17	155	-62.6028	0.044351
Bacteroides_400	1	121	384	-141.986	0.011798
Actinomyces_sp_oral_clone_DR002_406	1	18	114	109.3833	0.024171
Corynebacterium_propinquum_408	1	1	58	-39.9928	0.028154
Lachnospiraceae_419	1	205	384	162.215	0.044351
Staphylococcus_427	1	1	53	-24.6208	0.039201
Corynebacterium_freneyi_429	1	1	71	18.78578	0.038863
Coprobacter_443	1	54	205	-38.3623	0.02082
Bacteroides_445	1	1	384	-227.248	0.010182
Staphylococcaceae_452	1	1	57	28.1432	0.038863
Streptococcus_gallolyticus_subsp_macedonicus_464	1	1	20	-13.5917	0.008123
Escherichia_Shigella_469	1	1	164	-58.4555	0.028154
Bacilli_471	1	1	65	-43.2404	0.005386
Bifidobacterium_breve_473	1	1	60	-42.2703	0.008123
Bacteroides_482	1	213	384	-88.5204	0.017003
Plesiomonas_485	1	39	198	88.8588	0.015169
Bacteroides_493	1	56	227	-62.3357	0.026888
Veillonella_502	1	1	6	-2.77022	0.005386
Corynebacteriaceae_504	1	1	13	7.106628	0.010182
Corynebacteriaceae_504	2	38	122	-65.9096	0.017003
Staphylococcus_507	1	1	18	-11.9003	0.048666
Streptococcus_521	1	1	214	-79.5663	0.011798
Bacteroides_535	1	84	384	-88.7888	0.019389
Staphylococcus_565	1	1	22	-22.5613	0.005386
Ruminococcus_sp_CE2_596	1	105	384	-134.437	0.03052
Bacteroides_603	1	160	363	-68.0615	0.019389
Lachnospiraceae_614	1	136	384	-81.6236	0.046267
Lactobacillus_623	1	1	129	-20.4557	0.047473
Bifidobacterium_animalis_631	1	255	384	45.60759	0.03315
Bacteroides_645	1	1	149	38.29246	0.026888
Campylobacter_646	1	1	150	37.55262	0.033939
Bacteroides_657	1	1	285	-84.5281	0.005386
Bacteroides_658	1	63	384	-107.687	0.024171

Clostridium_sensu_stricto_1_673	1	60	188	26.37826	0.029147

Differential abundance testing by smoothing spline analysis of variance (SS-ANOVA) was executed to test in which specific intervals significant differences in OTUs existed between the groups, adjusted for mode of delivery and breastfeeding duration. The OTUs are arranged in descending order based on their relative abundance in this dataset. A positive Area value indicates that the abundance of a specific OTU is higher in the amoxicillin + cefotaxime regimen, while a negative area value indicates that the abundance of that OTU is higher in the controls. To correct for multiple testing, the Benjamini-Hochberg method was applied, and the adjusted p-values (p.adj) are shown. Int.no = interval number. In some cases, multiple OTUs of individual bacterial species were identified, so OTUs are referred to by their taxonomical annotations and a rank number (shown in parentheses), which is based on the abundance of each given OTU in the overall dataset.

Supplementary Table 11: Effect of antibiotic treatment in the first week of life on AMR gene composition in infants not receiving antibiotics later in life

**Time point**	**R^2 (%)**	**Adjusted p-value**
Before antibiotics	1.8	0.23
After antibiotics	7.0	0.001
Month 1	5.9	0.001
Month 4	2.7	0.001
Month 12	1.0	0.15

Effect sizes (R^2) and adjusted p-values are shown for the effect of antibiotics in the first week of life on AMR gene composition of the subset of participants not receiving antibiotics later in life. Source data are provided as a Source Data file.

Supplementary Table 12. Covariates significantly associated with AMR gene profile during follow-up

**Covariate**	**R^2 (%)**	**p.adj**
Age	4.9	0.001
Antibiotic treatment in 1st week of life (main study outcome)	2.1	0.001
Pets in the household	1.0	0.001
Mode of delivery	0.5	0.001
Breastfeeding at time of sampling	0.5	0.001
Gravidity	0.2	0.001
Siblings <5 years of age	0.2	0.001
Duration of ruptured membranes before birth	0.2	0.01

A temporal, multivariable permutational multivariate analysis of variance (PERMANOVA) test was performed with all covariates that were univariately associated with antimicrobial resistance (AMR) gene profile in cross-sectional analyses included in the model. The covariates that ensued as being associated with AMR gene profile during follow-up are shown here with their effect sizes (R^2) and adjusted p-values (p.adj, using Benjamini-Hochberg method to correct for multiple testing). Source data are provided as a Source Data file.

Supplementary Table 13: Effect of short (1-4 days) versus long (>4 days) antibiotic treatment in the first week of life on AMR gene composition

Time point	R^2 (%)	Adjusted p-value
Before antibiotics	1.4	0.92
After antibiotics	1.0	0.42
Month 1	0.8	0.52
Month 4	1.0	0.42
Month 12	1.2	0.42

Effect sizes (R^2) and adjusted p-values are shown for the effect of short versus long antibiotic treatment in the first week of life on AMR gene composition. Source data are provided as a Source Data file.

Supplementary Table 14: FitTimeSeries results of differentially abundant AMR genes between antibiotic treated infants and controls

Gene	Interval start	Interval end	Area	p.adj
aac6_aph2	1	140	-807	0.002
aac6_Ib	1	68	-35	0.04
aac6_Ii	1	185	-355	0.02
acrA	1	86	288	0.002
aph3_III	1	116	-205	0.03
blaAMPC	1	53	212	0.002
blaCMY_2	25	371	-423	0.02
blaCTX_M	1	196	252	0.03
blaNDM	1	180	216	0.02
blaTEM	1	112	-177	0.04
cblA	1	411	1085	0.004
ermB	1	213	-617	0.002
ermC	1	83	-263	0.002
MCR1	19	149	-73	0.002
mecA	1	147	-715	0.002
tetQ	1	306	1009	0.002

Differential abundance testing by smoothing spline analysis of variance (SS-ANOVA) was executed to test in which specific intervals significant differences in antimicrobial resistance (AMR) genes existed between the antibiotic treated infants and controls, adjusted for mode of delivery and siblings <5 years of age. Here, a negative Area value indicates that the abundance of a specific gene is higher in the antibiotic treated group, while a positive Area value indicates that the abundance of gene is higher in the controls. To correct for multiple testing, the Benjamini-Hochberg method was applied, and the adjusted p-values (p.adj) are shown.

Supplementary Table 15: List of significantly differentially abundant antimicrobial resistance (AMR) genes between amoxicillin + cefotaxime treated children and controls as studied by FitTimeSeries

Gene	Int.no	Interval start	Interval end	Area	p.adj
aac6_aph2	1	1	124	-815	0.0026
aac6_Ii	1	1	200	-536	0.0072
acrA	1	1	39	258	0.0026
aph3_Ia_Ic	1	32	53	-47	0.028
aph3_III	1	361	384	68	0.013
aph3_III	2	1	99	-229	0.029
blaAMPC	1	1	40	270	0.0026
blaCMY_2	1	25	139	-209	0.015
cblA	1	1	384	1283	0.0040
ermB	1	1	123	-494	0.0040
ermC	1	1	44	-225	0.0026
MCR1	1	95	153	-43	0.019
mecA	1	1	132	-822	0.0026
tetQ	1	1	316	1154	0.0026
vanA	1	1	223	-57	0.0026

Differential abundance testing by smoothing spline analysis of variance (SS-ANOVA) was executed to test in which specific intervals significant differences in AMR genes existed between children treated with amoxicillin + cefotaxime and controls. Here, a negative Area value indicates that the abundance of a specific gene is higher in the amoxicillin + cefotaxime group, while a positive Area value indicates that the abundance of gene is higher in the controls. To correct for multiple testing, the Benjamini-Hochberg method was applied, and the adjusted p-values (p.adj) are shown. Int.no = interval number.

Supplementary Table 16: List of significantly differentially abundant AMR genes between co-amoxiclav + gentamicin treated children and controls as studied by FitTimeSeries

Gene	Interval start	Interval end	Area	p.adj
aac6_aph2	1	312	-1615	0.0047
aac6_Ii	1	178	-442	0.012
aadE	199	411	373	0.031
aadE_like_gene	1	254	477	0.031
aph3_Ia_Ic	1	218	-396	0.027
aph3_III	1	130	-273	0.033
blaCMY_2	33	347	-470	0.027
blaCTX_M	1	166	249	0.033
blaNDM	2	191	241	0.027
blaTEM	1	259	-820	0.012
cblA	1	248	652	0.015
ermB	1	193	-528	0.011
ermC	1	160	-326	0.0047
MCR1	15	180	-151	0.012
mecA	1	162	-682	0.0047
tetQ	1	299	1187	0.0047

Differential abundance testing by smoothing spline analysis of variance (SS-ANOVA) was executed to test in which specific intervals significant differences in antimicrobial resistance (AMR) genes existed between children treated with co-amoxiclav + gentamicin and controls. Here, a negative Area value indicates that the abundance of a specific gene is higher in the co-amoxiclav + gentamicin, while a positive Area value indicates that the abundance of gene is higher in the controls. To correct for multiple testing, the Benjamini-Hochberg method was applied, and the adjusted p-values (p.adj) are shown.

Supplementary Table 17: List of significantly differentially abundant AMR genes between penicillin + gentamicin treated children and controls as studied by FitTimeSeries

Gene	Interval start	Interval end	Area	p.adj
aac6_aph2	1	141	-789	0.0026
acrA	1	73	234	0.0087
aph3_Ia_Ic	33	159	240	0.023
blaAMPC	1	72	247	0.0026
blaCMY_2	46	403	-394	0.028
blaCTX_M	1	179	317	0.0087
blaNDM	4	137	445	0.0026
cblA	31	223	338	0.028
ermB	1	182	-503	0.0087
ermC	1	88	-270	0.0026
mecA	1	169	-753	0.0026
tetQ	1	194	520	0.0087

Differential abundance testing by smoothing spline analysis of variance (SS-ANOVA) was executed to test in which specific intervals significant differences in antimicrobial resistance (AMR) genes existed between children treated with penicillin + gentamicin and controls. Here, a negative Area value indicates that the abundance of a specific gene is higher in the penicillin + gentamicin group, while a positive Area value indicates that the abundance of gene is higher in the controls. To correct for multiple testing, the Benjamini-Hochberg method was applied, and the adjusted p-values (p.adj) are shown.

Supplementary Table 18: List of differentially abundant AMR genes between amoxicillin + cefotaxime and co-amoxiclav + gentamicin treated children as studied by FitTimeSeries

Gene	Int.no	Interval start	Interval end	Area	p.adj
aadE	1	340	411	167	0.035
acrA	1	3	85	-342	0.021
aph2_Ide	3	280	364	5-12	0.035
aph2_Ide	4	28	36	-8E-14	0.032
aph2_Ide	5	129	253	-9E-12	0.035
blaAMPC	1	3	81	-343	0.021
blaKPC	1	345	411	93	0.036
blaTEM	1	3	355	-1292	0.021
ermB	1	3	18	57	0.032
ermC	1	3	23	60	0.032
mecA	1	3	62	153	0.035

Differential abundance testing by smoothing spline analysis of variance (SS-ANOVA) was executed to test in which specific intervals significant differences in antimicrobial resistance (AMR) genes existed between children treated with amoxicillin + cefotaxime and co-amoxiclav + gentamicin. Here, a negative Area value indicates that the abundance of a specific gene is higher in the co-amoxiclav + gentamicin group, while a positive Area value indicates that the abundance of gene is higher in the amoxicillin + cefotaxime group. To correct for multiple testing, the Benjamini-Hochberg method was applied, and the adjusted p-values (p.adj) are shown. Int.no = interval number.

Supplementary Table 19: List of differentially abundant AMR genes between amoxicillin + cefotaxime and penicillin + gentamicin treated children as observed by FitTimeSeries

Gene	Interval start	Interval end	Area	p.adj
aac6_Ii	2	157	384	0.042
acrA	2	24	-86	0.026
aph3_Ia_Ic	18	136	460	0.0070
blaAMPC	2	25	-84	0.026
blaNDM	2	17	21	0.042

Differential abundance testing by smoothing spline analysis of variance (SS-ANOVA) was executed to test in which specific intervals significant differences in antimicrobial resistance (AMR) genes existed between children treated with amoxicillin + cefotaxime and penicillin + gentamicin. Here, a negative Area value indicates that the abundance of a specific gene is higher in the penicillin + gentamicin group, while a positive Area value indicates that the abundance of gene is higher in the amoxicillin + cefotaxime group. To correct for multiple testing, the Benjamini-Hochberg method was applied, and the adjusted p-values (p.adj) are shown.

Supplementary Table 20: FitTimeSeries results of differentially abundant AMR genes between co-amoxiclav + gentamicin and penicillin + gentamicin treated children

Gene	Interval start	Interval end	Area	p.adj
aac3_Iiacde	2	191	577	0.030

Differential abundance testing by smoothing spline analysis of variance (SS-ANOVA) was executed to test in which specific intervals significant differences in antimicrobial resistance (AMR) genes existed between children treated with co-amoxiclav + gentamicin and penicillin + gentamicin. Here, a positive Area value indicates that the abundance of gene is higher in the co-amoxiclav + gentamicin group. To correct for multiple testing, the Benjamini-Hochberg method was applied, and the adjusted p-values (p.adj) are shown.

Supplementary Table 21: Significant correlations observed between OTUs and AMR genes

Gene	OTU	Pearson's r	p.adj
aac6_aph2	Bifidobacterium_1	0.42	0
aac6_IIa	Bifidobacterium_1	0.56	0.00012
ermC	Bifidobacterium_1	0.42	0
mecA	Bifidobacterium_1	0.55	0
acrA	Escherichia_coli_2	-0.65	0
aph2_Ib	Escherichia_coli_2	0.41	0.018
blaAMPC	Escherichia_coli_2	-0.69	0
blaTEM	Escherichia_coli_2	-0.51	0
vanB	Escherichia_coli_2	-0.42	0
aac6_aph2	Enterococcus_faecium_5	-0.42	0
aac6_Ii	Enterococcus_faecium_5	-0.50	0
aac6_IIa	Staphylococcus_epidermidis_3	-0.49	0.0026
ermC	Staphylococcus_epidermidis_3	-0.55	0
mecA	Staphylococcus_epidermidis_3	-0.58	0
blaCMY_2	Klebsiella_4	-0.40	0.0026
aac6_Ib	Clostridium_butyricum_35	-0.47	0.032
aac6_IIa	Lactobacillus_40	-0.47	0.0043
aac6_IIa	Corynebacterium_47	-0.45	0.0088
aac6_IIa	Erysipelotrichaceae_74	-0.46	0.0064
aac6_IIa	Tepidimonas_95	-0.50	0.0016
aac6_IIa	Comamonadaceae_130	-0.46	0.0056
aac6_IIa	Faecalibacterium_132	-0.46	0.0061
aac6_IIa	Blautia_141	-0.46	0.0064
aph2_Ide	Veillonella_115	-0.98	0.035
aac6_IIa	Ruminococcaceae_158	-0.46	0.0064
aac6_IIa	Lachnospiraceae_187	-0.46	0.0064
aac6_IIa	Bacteroides_314	-0.44	0.011
aac6_IIa	Corynebacterium_328	-0.46	0.0064
aac6_IIa	Lachnospiraceae_342	-0.46	0.0064
aac6_IIa	Coriobacteriaceae_377	-0.46	0.0064
aac6_IIa	Ruminococcaceae_387	-0.46	0.0064
aac6_IIa	Phascolarctobacterium_317	-0.46	0.0064
aac6_IIa	Bacteroides_coprophilus_402	-0.46	0.0064
aac6_IIa	Leuconostoc_mesenteroides_282	-0.43	0.013
aac6_IIa	Bacteroides_242	-0.46	0.0064
aac6_IIa	Oscillibacter_444	-0.46	0.0064
aac6_IIa	Ruminococcaceae_424	-0.46	0.0064
aac6_IIa	Prevotellaceae_449	-0.46	0.0064
aac6_IIa	Prevotella_509	-0.46	0.0064

aac6_IIa	Christensenellaceae_517	-0.46	0.0064
aac6_IIa	Anaerococcus_418	-0.46	0.0064
aac6_IIa	Faecalibacterium_477	-0.46	0.0064
aac6_IIa	Odoribacter_589	-0.46	0.0064
aac6_IIa	Staphylococcus_588	-0.41	0.025
aac6_IIa	Prevotella_558	-0.46	0.0064
aac6_Ib	Clostridium_sensu_stricto_1_621	-0.51	0.016
aac6_Ib	Clostridium_butyricum_685	-0.48	0.026

We correlated all 16S OTUs with all AMR genes found with the Fluidigm platform using the Pearson's correlation coefficient (two-sided). Depicted are all correlations with an adjusted p-value <0.05 and correlation coefficient of ≤-0.40 or ≥0.40. The OTUs are arranged in descending order based on their relative abundance in the overall dataset. A positive coefficient stands for a negative correlation between OTU and gene abundance, as a high Ct value indicates low abundance of an AMR gene. We applied the Benjamini-Hochberg method to correct for multiple testing and adjusted p-values (p.adj) are shown.

Supplementary Table 22: FitZig results of significantly differentially abundant AMR genes as found by MGS between antibiotic treated children and controls

Gene	log2FC	p.adj
Rif|CP003583.1|gene2745|Rifampin|Rifampin-resistant_beta-subunit_of_RNA_polymerase_RpoB|RPOB|RequiresSNPConfirmation	10.04607	1.71E-06
Flq|CP003583.1|gene1174|Fluoroquinolones|Fluoroquinolone-resistant_DNA_topoisomerases|PARC|RequiresSNPConfirmation	8.921015	1.21E-05
ACou|CP003583.1|gene1173|Aminocoumarins|Aminocoumarin-resistant_DNA_topoisomerases|PARE|RequiresSNPConfirmation	8.653329	1.48E-05
AGly|Aac6-Aph2|M13771|304-1743|1440|Aminoglycosides|Aminoglycoside_N-acetyltransferases|AAC6-PRIME	8.564101	3.08E-06
CARD|phgb|CP003583|2403484-2405254|ARO:3000776|adeC|Multi-drug_resistance|Multi-drug_efflux_pumps|ADEC	8.518329	2.18E-05
736|JX560992.1|JX560992|Aminoglycosides|Aminoglycoside_N-acetyltransferases|AAC6-PRIME	8.442804	5.2E-06
AGly|NC_005024.2598370|Aminoglycosides|Aminoglycoside_N-acetyltransferases|AAC6-PRIME	8.397573	5.2E-06
CARD|pvgb|CP003583|1050805-1052287|ARO:3003092|Enterococcus|Lipopeptides|Daptomycin-resistant_cls|CLS|RequiresSNPConfirmation	8.274826	2.65E-05
MLS|MsrC|AY004350|496-1974|1479|MLS|Macrolide_resistance_efflux_pumps|MSRC	8.017561	2.45E-05
Rif|AE016830.1|gene3155|Rifampin|Rifampin-resistant_beta-subunit_of_RNA_polymerase_RpoB|RPOB|RequiresSNPConfirmation	7.921589	1.11E-05
CARD|pvgb|CP003583|913745-914813|ARO:3003079|Enterococcus|Lipopeptides|Daptomycin-resistant_liaFSR|LIAFSR|RequiresSNPConfirmation	7.66265	5.43E-05
Flq|AE016830.1|gene6|Fluoroquinolones|Fluoroquinolone-resistant_DNA_topoisomerases|GYRA|RequiresSNPConfirmation	7.519387	1.45E-05
CARD|pvgb|HE999704|2567249-2568437|ARO:3001312|elfamycin|Elfamycins|EF-Tu_inhibition|TUFAB|RequiresSNPConfirmation	7.403174	1.43E-05
CARD|pvgb|NC_007622|559336-560521|ARO:3001312|elfamycin|Elfamycins|EF-Tu_inhibition|TUFAB|RequiresSNPConfirmation	7.368328	9.39E-08
940|AF028812.1|AF028812|Trimethoprim|Dihydrofolate_reductase|DFRF	7.329183	4.98E-05
Elf|NC_007622.3795131|Elfamycins|EF-Tu_inhibition|TUFAB|RequiresSNPConfirmation	7.206572	1.31E-07
Tet|tetM_13_AM990992|Tetracyclines|Tetracycline_resistance_ribosomal_protection_proteins|TETM	7.163641	2.45E-05
Rif|NC_003098.1.933647|Rifampin|Rifampin-resistant_beta-subunit_of_RNA_polymerase_RpoB|RPOB|RequiresSNPConfirmation	7.145893	1.74E-06
Elf|HE999704.1|gene2984|Elfamycins|EF-Tu_inhibition|TUFAB|RequiresSNPConfirmation	7.124943	1.48E-05
Flq|AE015929.1|gene5|Fluoroquinolones|Fluoroquinolone-resistant_DNA_topoisomerases|GYRA|RequiresSNPConfirmation	7.073008	2.65E-05
Elf|NC_002745.1123311|Elfamycins|EF-Tu_inhibition|TUFAB|RequiresSNPConfirmation	7.02243	1.31E-07
CARD|pvgb|NC_002952|594518-598142|ARO:3003291|Staphylococcus|Lipopeptides|Daptomycin-resistant_beta-subunit_of_RNA_polymerase_RpoC|RPOC|RequiresSNPConfirmation	6.978523	1.21E-05
Tet|tetM_8_X04388|Tetracyclines|Tetracycline_resistance_ribosomal_protection_proteins|TETM	6.974866	3.37E-05
Elf|NC_002951.3236433|Elfamycins|EF-Tu_inhibition|TUFAB|RequiresSNPConfirmation	6.872335	5.77E-07
CARD|pvgb|AP009048|3760295-3762710|ARO:3003303|Escherichia|Fluoroquinolones|Fluoroquinolone-resistant_DNA_topoisomerases|GYRB|RequiresSNPConfirmation	6.807278	5.61E-05
Tet|tetM_6_M21136|Tetracyclines|Tetracycline_resistance_ribosomal_protection_proteins|TETM	6.777552	4.4E-05
Mdr|AB566411.1|gene1|Multi-drug_resistance|Multi-drug_efflux_pumps|QACAB	6.749747	9.71E-05
Rif|NC_012469.1.7686402|Rifampin|Rifampin-resistant_beta-subunit_of_RNA_polymerase_RpoB|RPOB|RequiresSNPConfirmation	6.738756	3.4E-06
CARD|pvgb|NC_003197|4038854-4041269|ARO:3003307|Salmonella|Fluoroquinolones|Fluoroquinolone-resistant_DNA_topoisomerases|GYRB|RequiresSNPConfirmation	6.698354	6.98E-05
Rif|CP000647.1|gene4402|Rifampin|Rifampin-resistant_beta-subunit_of_RNA_polymerase_RpoB|RPOB|RequiresSNPConfirmation	6.691371	5.61E-05
Elf|NC_002952.2860408|Elfamycins|EF-Tu_inhibition|TUFAB|RequiresSNPConfirmation	6.568222	3.92E-06
Bla|mecA_15_AB505628|betalactams|Penicillin_binding_protein|MECA	6.486463	0.0002
Elf|CP000647.1|gene4394|Elfamycins|EF-Tu_inhibition|TUFAB|RequiresSNPConfirmation	6.463796	5.61E-05
Tet|tetM_1_X92947|Tetracyclines|Tetracycline_resistance_ribosomal_protection_proteins|TETM	6.358008	9.56E-05
Rif|FN543093.2|gene314|Rifampin|Rifampin-resistant_beta-subunit_of_RNA_polymerase_RpoB|RPOB|RequiresSNPConfirmation	6.320669	5.43E-05

Rif|NC_009487.5169226|Rifampin|Rifampin-resistant_beta-subunit_of_RNA_polymerase_RpoB|RPOB|RequiresSNPConfirmation	6.312594	7.68E-05
605|V01547.1|V01547|Aminoglycosides|Aminoglycoside_O-phosphotransferases|APH3-PRIME	6.309507	0.000322
CARD|pvgb|AE014075|3901532-3902762|ARO:3003438|Escherichia|Elfamycins|EF-Tu_inhibition|TUFAB|RequiresSNPConfirmation	6.279452	0.000207
CARD|phgb|NC_012469|1800927-1802391|ARO:3000616|mel|MLS|Macrolide_resistance_efflux_pumps|MEL	6.272894	2.45E-05
CARD|phgb|AB037671|24420-26427|ARO:3001209|mecC|betalactams|Penicillin_binding_protein|MECC	6.269044	0.000247
Flq|CP000647.1|gene3437|Fluoroquinolones|Fluoroquinolone-resistant_DNA_topoisomerases|PARC|RequiresSNPConfirmation	6.263472	0.000969
CARD|pvgb|CP003583|914819-915452|ARO:3003078|Enterococcus|Lipopeptides|Daptomycin-resistant_liaFSR|LIAFSR|RequiresSNPConfirmation	6.217033	0.000793
Flq|AE016830.1|gene1598|Fluoroquinolones|Fluoroquinolone-resistant_DNA_topoisomerases|PARC|RequiresSNPConfirmation	6.192162	0.000217
Bla|KC243783.1|gene1|betalactams|Penicillin_binding_protein|MECA	6.163537	0.000249
Tet|tetM_7_FN433596|Tetracyclines|Tetracycline_resistance_ribosomal_protection_proteins|TETM	6.143436	0.000142
1463|M81802.1|STAMSRB|MLS|Macrolide_resistance_efflux_pumps|MSRA	6.141506	0.000306
Flq|OqxBgb|EU370913|47851-51003|3153|Multi-drug_resistance|Multi-drug_efflux_pumps|OQXB	6.123797	0.000945
CARD|phgb|AB091338|173-1355|ARO:3003551|emeA|Multi-drug_resistance|Multi-drug_efflux_pumps|EMEA	6.117836	0.000243
MLS|ermB_6_AF242872|MLS|23S_rRNA_methyltransferases|ERMB	6.103928	0.000534
Flq|CP001918.1|gene3562|Fluoroquinolones|Fluoroquinolone-resistant_DNA_topoisomerases|GYRA|RequiresSNPConfirmation	6.072613	0.001581
Bla|AF336096.1|gene1|betalactams|Mutant_porin_proteins|OMP36|RequiresSNPConfirmation	6.061827	1.5E-05
Bla|NC_002745.1122813|betalactams|Penicillin_binding_protein|MECA	6.048143	0.000249
CARD|phgb|L12710|0-549|ARO:3002556|AAC6-PRIME-Ii|Aminoglycosides|Aminoglycoside_N-acetyltransferases|AAC6-PRIME	6.048094	0.000916
Flq|CP001138.1|gene2385|Fluoroquinolones|Fluoroquinolone-resistant_DNA_topoisomerases|GYRA|RequiresSNPConfirmation	5.951057	0.000348
Bla|NC_009487.5167689|betalactams|Penicillin_binding_protein|MECA	5.949511	0.000422
Mdr|oqxB_1_EU370913|Multi-drug_resistance|Multi-drug_efflux_pumps|OQXB	5.947138	0.001235
Mdr|AY661734.1|gene2|Multi-drug_resistance|Multi-drug_efflux_pumps|MEPA	5.937923	0.011589
Elf|CP000647.1|gene3761|Elfamycins|EF-Tu_inhibition|TUFAB|RequiresSNPConfirmation	5.934923	0.000138
11|DQ679966.1|DQ679966|Multi-drug_resistance|Multi-drug_efflux_pumps|ACRB	5.933073	0.000348
MLS|ermB_15U48430|MLS|23S_rRNA_methyltransferases|ERMB	5.915585	0.000533
Bla|AF336097.1|gene1|betalactams|Mutant_porin_proteins|OMP36|RequiresSNPConfirmation	5.89168	0.000207
Flq|CP001138.1|gene3329|Fluoroquinolones|Fluoroquinolone-resistant_DNA_topoisomerases|PARC|RequiresSNPConfirmation	5.881479	0.001782
MLS|MsrD|AF274302|2462-3925|1464|MLS|Macrolide_resistance_efflux_pumps|MSRD	5.88022	3.52E-05
Tet|tetM_12_FR671418|Tetracyclines|Tetracycline_resistance_ribosomal_protection_proteins|TETM	5.87791	0.000249
Tet|tetM_5_U58985|Tetracyclines|Tetracycline_resistance_ribosomal_protection_proteins|TETM	5.859626	0.000312
Flq|FN543093.2|gene2948|Fluoroquinolones|Fluoroquinolone-resistant_DNA_topoisomerases|GYRA|RequiresSNPConfirmation	5.857616	0.001913
ACou|CP000647.1|gene3444|Aminocoumarins|Aminocoumarin-resistant_DNA_topoisomerases|PARE|RequiresSNPConfirmation	5.816394	0.001497
ACou|AE016830.1|gene1599|Aminocoumarins|Aminocoumarin-resistant_DNA_topoisomerases|PARE|RequiresSNPConfirmation	5.816186	0.000457
CARD|phgb|U00096|2155262-2158385|ARO:3000793|mdtB|Multi-drug_resistance|Multi-drug_efflux_pumps|MDTB	5.768583	0.000312
Sul|AE016830.1|gene3181|Sulfonamides|Sulfonamide-resistant_dihydropteroate_synthases|FOLP|RequiresSNPConfirmation	5.760223	0.000797
Rif|CP000034.1|gene3741|Rifampin|Rifampin-resistant_beta-subunit_of_RNA_polymerase_RpoB|RPOB|RequiresSNPConfirmation	5.688878	0.000312
MLS|LsaA|AY225127|41-1537|1497|MLS|Streptogramin_resistance_ATP-binding_cassette_ABC_efflux_pumps|LSA	5.672168	0.000536
MLS|mefA_10_AF376746|MLS|Macrolide_resistance_efflux_pumps|MEFA	5.66807	8.54E-05
Tet|tetM_2_X90939|Tetracyclines|Tetracycline_resistance_ribosomal_protection_proteins|TETM	5.653387	0.000249

Tet|tetM_11_JN846696|Tetracyclines|Tetracycline_resistance_ribosomal_protection_proteins|TETM	5.638708	0.000109
CARD|phgb|U00096|2812615-2814154|ARO:3000074|emrB|Multi-drug_resistance|Multi-drug_efflux_pumps|EMRB	5.638329	0.000275
Rif|CP001918.1|gene250|Rifampin|Rifampin-resistant_beta-subunit_of_RNA_polymerase_RpoB|RPOB|RequiresSNPConfirmation	5.634411	0.000945
CARD|phgb|NC_007779|2586250-2589364|ARO:3000491|acrD|Aminoglycosides|Aminoglycoside_efflux_pumps|ACRD	5.598443	0.000457
1021|AJ243209.1|AJ243209|MLS|Macrolide_resistance_efflux_pumps|MSRC	5.597685	0.000793
23|U58210.1|STU58210|betalactams|Penicillin_binding_protein|PBP2B	5.544856	4.4E-05
Elf|CP001918.1|gene242|Elfamycins|EF-Tu_inhibition|TUFAB|RequiresSNPConfirmation	5.520286	0.000945
CARD|pvgb|AE016830|2790820-2791552|ARO:3003077|Enterococcus|Lipopeptides|Daptomycin-resistant_liaFSR|LIAFSR|RequiresSNPConfirmation	5.490529	0.000679
Rif|NC_007622.3792928|Rifampin|Rifampin-resistant_beta-subunit_of_RNA_polymerase_RpoB|RPOB|RequiresSNPConfirmation	5.487949	0.000519
Rif|AP009048.1|gene3341|Rifampin|Rifampin-resistant_beta-subunit_of_RNA_polymerase_RpoB|RPOB|RequiresSNPConfirmation	5.470726	0.000681
MLS|lsaA_3_AY737526|MLS|Streptogramin_resistance_ATP-binding_cassette_ABC_efflux_pumps|LSA	5.419232	0.00054
Flq|FN543093.2|gene3588|Fluoroquinolones|Fluoroquinolone-resistant_DNA_topoisomerases|PARC|RequiresSNPConfirmation	5.382396	0.003009
Bla|mecA_7_BA000018|betalactams|Penicillin_binding_protein|MECA	5.377954	0.000533
Bla|blaZ_34_AP003139|betalactams|Class_A_betalactamases|BLAZ	5.342823	0.000237
Bla|AF336095.1|gene1|betalactams|Mutant_porin_proteins|OMP36|RequiresSNPConfirmation	5.339979	0.000312
CARD|pvgb|CP000647|4764664-4765849|ARO:3001312|elfamycin|Elfamycins|EF-Tu_inhibition|TUFAB|RequiresSNPConfirmation	5.337423	0.000249
Tet|TetM|U08812|1981-3900|1920|Tetracyclines|Tetracycline_resistance_ribosomal_protection_proteins|TETM	5.29587	0.000793
Flq|CP000647.1|gene2640|Fluoroquinolones|Fluoroquinolone-resistant_DNA_topoisomerases|GYRA|RequiresSNPConfirmation	5.293169	0.001025
Bla|MECA|AB221124|91-2097|2007|betalactams|Penicillin_binding_protein|MECA	5.290229	0.000818
1454|J01764.1|PT1CG|Tetracyclines|Tetracycline_resistance_major_facilitator_superfamily_MFS_efflux_pumps|TETK	5.288726	0.002392
Tet|tetM_4_X75073|Tetracyclines|Tetracycline_resistance_ribosomal_protection_proteins|TETM	5.248432	0.000716
Rif|CP001138.1|gene4362|Rifampin|Rifampin-resistant_beta-subunit_of_RNA_polymerase_RpoB|RPOB|RequiresSNPConfirmation	5.221324	0.00094
Tet|tetM_9_X56353|Tetracyclines|Tetracycline_resistance_ribosomal_protection_proteins|TETM	5.212076	0.000614
AGly|AY712687.1|gene1|Aminoglycosides|Aminoglycoside_O-nucleotidyltransferases|ANT6	5.207654	0.001759
CARD|pvgb|U00096|3163714-3165973|ARO:3003308|Escherichia|Fluoroquinolones|Fluoroquinolone-resistant_DNA_topoisomerases|PARC|RequiresSNPConfirmation	5.190758	0.001405
179|M16217.1|PNS1CG|Tetracyclines|Tetracycline_resistance_major_facilitator_superfamily_MFS_efflux_pumps|TETK	5.178832	0.004669
CARD|phgb|AM180355|2319373-2320111|ARO:3000375|ErmB|MLS|23S_rRNA_methyltransferases|ERM	5.170926	0.000716
CARD|pvgb|CP001918|235165-236350|ARO:3001312|elfamycin|Elfamycins|EF-Tu_inhibition|TUFAB|RequiresSNPConfirmation	5.160506	0.001913
Bla|mecA_10_AB512767|betalactams|Penicillin_binding_protein|MECA	5.159533	0.000298
AGly|Ant6-Ia|AF330699|22-930|909|Aminoglycosides|Aminoglycoside_O-nucleotidyltransferases|ANT6	5.142961	0.001268
Bla|AmpH|CP003785|4208384-4209544|1161|betalactams|Penicillin_binding_protein|AMPH	5.125804	0.003508
CARD|pvgb|CP001918|4816613-4817537|ARO:3001312|elfamycin|Elfamycins|EF-Tu_inhibition|TUFAB|RequiresSNPConfirmation	5.111733	0.000768
Flq|NC_003197.1.1253794|Fluoroquinolones|Fluoroquinolone-resistant_DNA_topoisomerases|GYRA|RequiresSNPConfirmation	5.107397	0.001759
Bla|NC_010066.5774791|betalactams|Class_A_betalactamases|BLAZ	5.088592	0.000237
Rif|NC_007793.3914836|Rifampin|Rifampin-resistant_beta-subunit_of_RNA_polymerase_RpoB|RPOB|RequiresSNPConfirmation	5.079887	0.000207
Rif|NC_002745.1123305|Rifampin|Rifampin-resistant_beta-subunit_of_RNA_polymerase_RpoB|RPOB|RequiresSNPConfirmation	5.074613	0.000519
MLS|ermB_11_M19270|MLS|23S_rRNA_methyltransferases|ERMB	5.033314	0.000922
Elf|CP001918.1|gene4764|Elfamycins|EF-Tu_inhibition|TUFAB|RequiresSNPConfirmation	5.022946	0.001619
Flq|NC_003197.1.1254697|Fluoroquinolones|Fluoroquinolone-resistant_DNA_topoisomerases|PARC|RequiresSNPConfirmation	5.020705	0.00428

Tet|tetM_10_EU182585|Tetracyclines|Tetracycline_resistance_ribosomal_protection_proteins|TETM	4.96337	0.000573
MLS|ermC_13_M13761|MLS|23S_rRNA_methyltransferases|ERMC	4.948993	0.000945
738|JX560992.1|JX560992|Aminoglycosides|Aminoglycoside_O-nucleotidyltransferases|ANT6	4.931574	0.00224
Bla|mecA_4_AB033763|betalactams|Penicillin_binding_protein|MECA	4.927409	0.000916
Bla|OXY6-2|AJ871875|1-873|873|betalactams|Class_A_betalactamases|OXY	4.927085	0.00128
MLS|mphC_1_AB013298|MLS|Macrolide_phosphotransferases|MPHC	4.862321	0.001963
MLS|ermB_9_AF299292|MLS|23S_rRNA_methyltransferases|ERMB	4.839503	0.002544
Flq|M58408|gene|Fluoroquinolones|Fluoroquinolone-resistant_DNA_topoisomerases|PARC|RequiresSNPConfirmation	4.83048	0.001782
Bla|OXY6-4|AJ871877|1-873|873|betalactams|Class_A_betalactamases|OXY	4.82262	0.003385
MLS|lsaA_2_AY58982|MLS|Streptogramin_resistance_ATP-binding_cassette_ABC_efflux_pumps|LSA	4.776619	0.002408
AGly|Sat4A|X92945|38870-39412|543|Aminoglycosides|Aminoglycoside_N-acetyltransferases|SAT	4.750136	0.003009
CARD|phgb|NC_010066|9682-10528|ARO:3000621|PC1|betalactams|Class_A_betalactamases|BLAZ	4.744185	0.000861
CARD|pvgb|NC_002952|590830-594382|ARO:3003287|Staphylococcus|Lipopeptides|Daptomycin-resistant_beta-subunit_of_RNA_polymerase_RpoB|rpoB|RequiresSNPConfirmation	4.732193	0.000772
gi|149167|gb|M88143.1|KPNBETALAC|betalactams|Class_A_betalactamases|TEM	4.730381	0.001782
CARD|phgb|NC_023287|60996-61860|ARO:3002626|ANT6-Ia|Aminoglycosides|Aminoglycoside_O-nucleotidyltransferases|ANT6	4.726548	0.003919
CARD|phgb|AF028811|0-462|ARO:3002875|dfrE|Trimethoprim|Dihydrofolate_reductase|DFRE	4.719341	0.001778
199|AY566250.1|AY566250|Fluoroquinolones|Quinolone_active_efflux|NORA	4.715596	0.003745
CARD|phgb|NC_002695|1737553-1737967|ARO:3000676|H-NS|Multi-drug_resistance|MDR_regulator|HNS	4.697152	0.000818
CARD|phgb|U00096|3415032-3418137|ARO:3000502|acrF|Aminoglycosides|Aminoglycoside_efflux_pumps|ACRF	4.679166	0.003379
Fos|fosA_8_ACHE01000077|Fosfomycin|Fosfomycin_thiol_transferases|FOSA	4.657483	0.001497
AGly|APH-Stph|HE579073|1778413-1779213|801|Aminoglycosides|Aminoglycoside_O-phosphotransferases|APH3-PRIME	4.657181	0.001633
CARD|phgb|U00096|2158385-2161463|ARO:3000794|mdtC|Multi-drug_resistance|Multi-drug_efflux_pumps|MDTC	4.65583	0.001997
CARD|pvgb|NC_002695|4990140-4994169|ARO:3003288|Escherichia|Rifampin|Rifampin-resistant_beta-subunit_of_RNA_polymerase_RpoB|RPOB|RequiresSNPConfirmation	4.645964	0.001268
246|DQ679966.1|DQ679966|Multi-drug_resistance|Multi-drug_efflux_pumps|MEXE	4.626894	0.002908
1066|unknown_id|unknown_name|MLS|23S_rRNA_methyltransferases|ERMB	4.608049	0.003764
MLS|ermB_12_U18931|MLS|23S_rRNA_methyltransferases|ERMB	4.603798	0.004169
CARD|phgb|AP009048|4153663-4154296|ARO:3000518|CRP|Multi-drug_resistance|MDR_regulator|CRP	4.601957	0.001127
Flq|NC_012469.1.7686721|Fluoroquinolones|Fluoroquinolone-resistant_DNA_topoisomerases|GYRA|RequiresSNPConfirmation	4.589629	3.37E-05
Bla|X04121.1|gene1|betalactams|Class_A_betalactamases|BLAZ	4.558406	0.001759
Rif|NC_002951.3236234|Rifampin|Rifampin-resistant_beta-subunit_of_RNA_polymerase_RpoB|RPOB|RequiresSNPConfirmation	4.550078	0.00071
CARD|pvgb|NC_007779|3172159-3174052|ARO:3003316|Escherichia|Fluoroquinolones|Fluoroquinolone-resistant_DNA_topoisomerases|parE|RequiresSNPConfirmation	4.538684	0.001997
554|FN806789.1|FN806789|MLS|23S_rRNA_methyltransferases|ERMB	4.527378	0.003248
Rif|NC_003197.1.1255679|Rifampin|Rifampin-resistant_beta-subunit_of_RNA_polymerase_RpoB|RPOB|RequiresSNPConfirmation	4.505091	0.001633
MLS|msrA_2_AB013298|MLS|Macrolide_resistance_efflux_pumps|MSRA	4.479036	0.009354
CARD|phgb|U00096|3660413-3663527|ARO:3000796|mdtF|Multi-drug_resistance|Multi-drug_efflux_pumps|MDTC	4.465299	0.005238
Rif|NC_002695.1.914942|Rifampin|Rifampin-resistant_beta-subunit_of_RNA_polymerase_RpoB|RPOB|RequiresSNPConfirmation	4.445286	0.002337
Tet|tetW_1_DQ060146|Tetracyclines|Tetracycline_resistance_ribosomal_protection_proteins|TETW	4.436131	0.022933
AGly|aph3-prime-Ia_1_V00359|Aminoglycosides|Aminoglycoside_O-phosphotransferases|APH3-PRIME	4.427153	0.003692
Bla|mecA_14_AB505630|betalactams|Penicillin_binding_protein|MECA	4.427001	0.00269
Mdr|NC_002695.1.910541|Multi-drug_resistance|MDR_regulator|CPXAR	4.423297	0.004103

Bla|blaZ_40_DQ269019|betalactams|Class_A_betalactamases|BLAZ	4.42069	0.000629
Rif|NC_013450.8613267|Rifampin|Rifampin-resistant_beta-subunit_of_RNA_polymerase_RpoB|RPOB|RequiresSNPConfirmation	4.418607	0.001099
CARD|pvgb|FQ312006|705198-706383|ARO:3001312|elfamycin|Elfamycins|EF-Tu_inhibition|TUFAB|RequiresSNPConfirmation	4.417459	0.004487
MLS|ermB_17_X64695|MLS|23S_rRNA_methyltransferases|ERMB	4.401039	0.004573
Tet|tetW_6_FN396364|Tetracyclines|Tetracycline_resistance_ribosomal_protection_proteins|TETW	4.392852	0.004103
CARD|pvgb|NC_002952|1419759-1422162|ARO:3003312|Staphylococcus|Fluoroquinolones|Fluoroquinolone-resistant_DNA_topoisomerases|PARC|RequiresSNPConfirmation	4.348429	0.004077
13|L11616.1|PSEENVCD|Multi-drug_resistance|Multi-drug_efflux_pumps|MEXB	4.324791	0.001266
Bla|PBP|Ecoli|CP002291|664439-666340|1902|betalactams|Penicillin_binding_protein|PBP2	4.316706	0.00493
MLS|ermB_1_JN899585|MLS|23S_rRNA_methyltransferases|ERMB	4.31464	0.004104
Flq|OqxA|EU370913|46652-47827|1176|Multi-drug_resistance|Multi-drug_efflux_pumps|OQXA	4.30986	0.017512
AGly|U01945.1|gene1|Aminoglycosides|Aminoglycoside_N-acetyltransferases|SAT	4.286232	0.006611
CARD|phgb|U00096|2484373-2487967|ARO:3000833|evgS|Multi-drug_resistance|MDR_regulator|EVGS	4.257267	0.006704
MLS|MphC|AF167161|5665-6564|900|MLS|Macrolide_phosphotransferases|MPHC	4.240403	0.003919
CARD|phgb|AE015929|1128933-1129419|ARO:3002865|dfrC|Trimethoprim|Dihydrofolate_reductase|DFRC|RequiresSNPConfirmation	4.221985	0.003666
Bla|BlaZ|AB245469|2235-3080|861|betalactams|Class_A_betalactamases|BLAZ	4.212092	0.005429
CARD|phgb|CP000034|2662758-2663289|ARO:3000516|emrR|Multi-drug_resistance|MDR_regulator|EMRR	4.207096	0.006385
Mdr|AF535087.1|gene1|Multi-drug_resistance|Multi-drug_efflux_pumps|QACAB	4.206097	0.008229
Mdr|AP009048.1|gene2135|Multi-drug_resistance|Multi-drug_efflux_pumps|MDTD	4.197468	0.004173
CARD|pvgb|NC_002952|590830-594382|ARO:3003285|Staphylococcus|Rifampin|Rifampin-resistant_beta-subunit_of_RNA_polymerase_RpoB|RPOB|RequiresSNPConfirmation	4.143949	0.001782
Bla|Z_35_AJ302698|betalactams|Class_A_betalactamases|BLAZ	4.12885	0.005563
MLS|ermB_2_K00551|MLS|23S_rRNA_methyltransferases|ERMB	4.127583	0.005999
CARD|pvgb|AL450380|2273268-2276805|ARO:3003284|Mycobacterium|Rifampin|Rifampin-resistant_beta-subunit_of_RNA_polymerase_RpoB|RPOB|RequiresSNPConfirmation	4.125903	0.017476
CARD|phgb|AY047358|1710-2352|ARO:3003552|fusB|Fusidic_acid|Ribosomal_zinc-binding_protein|FUSB	4.07809	0.00478
Rif|NC_002758.1120515|Rifampin|Rifampin-resistant_beta-subunit_of_RNA_polymerase_RpoB|RPOB|RequiresSNPConfirmation	4.077947	0.001997
672|AF181950.1|AF181950|betalactams|Penicillin_binding_protein|MECA	4.070819	0.001468
MLS|ermB_18_X66468|MLS|23S_rRNA_methyltransferases|ERMB	4.064816	0.005741
Bla|AF336098.1|gene1|betalactams|Mutant_porin_proteins|OMP36|RequiresSNPConfirmation	4.057089	0.003132
ACou|NC_003197.1.1254704|Aminocoumarins|Aminocoumarin-resistant_DNA_topoisomerases|PARE|RequiresSNPConfirmation	4.04722	0.048887
ACou|CP000034.1|gene3210|Aminocoumarins|Aminocoumarin-resistant_DNA_topoisomerases|PARE|RequiresSNPConfirmation	4.006142	0.002408
Flq|NC_003098.1.933198|Fluoroquinolones|Fluoroquinolone-resistant_DNA_topoisomerases|GYRA|RequiresSNPConfirmation	3.990149	0.000247
CARD|pvgb|CP000647|489264-489915|ARO:3003373|Klebsiella|Multi-drug_resistance|MDR_regulator|ACRR|RequiresSNPConfirmation	3.949866	0.007895
Tet|tetO_3_Y07780|Tetracyclines|Tetracycline_resistance_ribosomal_protection_proteins|TETO	3.932456	0.00259
Bla|AY077483.1|gene1|betalactams|Class_A_betalactamases|OXY	3.926918	0.016
Bla|Z_32_AP004832|betalactams|Class_A_betalactamases|BLAZ	3.922653	0.002746
Bla|NC_010063.5774822|betalactams|Class_A_betalactamases|BLAZ	3.921497	0.000888
Flq|NC_003098.1.934295|Fluoroquinolones|Fluoroquinolone-resistant_DNA_topoisomerases|PARC|RequiresSNPConfirmation	3.896854	0.000237
252|FJ744595.1|FJ744595|Multi-drug_resistance|Multi-drug_efflux_pumps|EMRD	3.885592	0.018497
Sul|CP000647.1|gene3624|Sulfonamides|Sulfonamide-resistant_dihydropteroate_synthases|FOLP|RequiresSNPConfirmation	3.866427	0.014305
CARD|phgb|AP009048|2810082-2811255|ARO:3000027|emrA|Multi-drug_resistance|Multi-drug_efflux_pumps|EMRA	3.866067	0.008436

ACou|FN543093.2|gene3601|Aminocoumarins|Aminocoumarin-resistant_DNA_topoisomerases|PARE|RequiresSNPConfirmation	3.866029	0.016082
MLS|ermB_7_AF368302|MLS|23S_rRNA_methyltransferases|ERMB	3.865604	0.008653
1268|AB976602.1|AB976602|betalactams|Class_A_betalactamases|CTX	3.822986	0.0145
Flq|NC_002952.2859941|Fluoroquinolones|Fluoroquinolone-resistant_DNA_topoisomerases|PARC|RequiresSNPConfirmation	3.808601	0.004573
MLS|msrA_1_X52085|MLS|Macrolide_resistance_efflux_pumps|MSRA	3.778458	0.00578
CARD|phgb|AP009048|4304505-4306557|ARO:3003549|mdtO|Multi-drug_resistance|Multi-drug_efflux_pumps|MDTO	3.773864	0.016082
Mdr|AP009048.1|gene3415|Multi-drug_resistance|MDR_regulator|CPXAR	3.766044	0.013282
MLS|msrC_2_AF313494|MLS|Macrolide_resistance_efflux_pumps|MSRC	3.757821	0.008803
CARD|pvgb|AF335467|36-1164|ARO:3003385|Enterobacter|Multi-drug_resistance|MDR_mutant_porin_proteins|omp36|RequiresSNPConfirmation	3.751259	0.010853
Sul|NC_012469.1.7686560|Sulfonamides|Sulfonamide-resistant_dihydropteroate_synthases|FOLP|RequiresSNPConfirmation	3.744433	0.000749
1737|AY033516.1|AY033516|betalactams|Class_A_betalactamases|CTX	3.740823	0.009108
CARD|phgb|NC_003098|1886035-1888501|ARO:3003044|PBP1b|betalactams|Penicillin_binding_protein|PBP1B	3.702681	0.000888
CARD|phgb|AP009048|4338624-4340268|ARO:3003576|PmrC|Cationic_antimicrobial_peptides|Lipid_A_modification|PMRC	3.691995	0.017673
CARD|pvgb|U00096|2336792-2339420|ARO:3003294|Escherichia|Fluoroquinolones|Fluoroquinolone-resistant_DNA_topoisomerases|GYRA|RequiresSNPConfirmation	3.675845	0.011589
Flq|NC_012469.1.7685406|Fluoroquinolones|Fluoroquinolone-resistant_DNA_topoisomerases|PARC|RequiresSNPConfirmation	3.663717	0.00016
CARD|phgb|U00096|3203309-3204131|ARO:3002986|bacA|Bacitracin|Undecaprenyl_pyrophosphate_phosphatase|BACA	3.662531	0.009354
Elf|FQ312006.1|gene760|Elfamycins|EF-Tu_inhibition|TUFAB|RequiresSNPConfirmation	3.651592	0.013375
ACou|NC_003098.1.934298|Aminocoumarins|Aminocoumarin-resistant_DNA_topoisomerases|PARE|RequiresSNPConfirmation	3.644373	0.000243
Tmt|DfrC|Z48233|337-822|486|Trimethoprim|Dihydrofolate_reductase|DFRC|RequiresSNPConfirmation	3.642526	0.006927
CARD|phgb|U00096|2368038-2370021|ARO:3002985|arnA|Cationic_antimicrobial_peptides|Lipid_A_modification|ARNA	3.62985	0.017612
CARD|pvgb|AE005672|800699-803171|ARO:3003311|Streptococcus|Fluoroquinolones|Fluoroquinolone-resistant_DNA_topoisomerases|PARC|RequiresSNPConfirmation	3.617412	0.000242
MLS|MsrA|AY591760|274-1740|1467|MLS|Macrolide_resistance_efflux_pumps|MSRA	3.615451	0.016082
Flq|NC_002952.2859949|Fluoroquinolones|Fluoroquinolone-resistant_DNA_topoisomerases|GYRA|RequiresSNPConfirmation	3.609137	0.033068
Bla|AmpC1_Ecoli|FN649414|2765051-2766355|1302|betalactams|Penicillin_binding_protein|PBP4B	3.602782	0.026057
Flq|CP000034.1|gene2423|Fluoroquinolones|Fluoroquinolone-resistant_DNA_topoisomerases|GYRA|RequiresSNPConfirmation	3.591779	0.016082
Mdr|CP000034.1|gene2198|Multi-drug_resistance|MDR_regulator|ASMA	3.555453	0.020306
Rif|NC_002953.2862188|Rifampin|Rifampin-resistant_beta-subunit_of_RNA_polymerase_RpoB|RPOB|RequiresSNPConfirmation	3.554997	0.004103
CARD|phgb|NC_007779|1745170-1746544|ARO:3001327|mdtK|Multi-drug_resistance|Multi-drug_efflux_pumps|MDTK	3.540711	0.014911
MLS|ermB_16_X82819|MLS|23S_rRNA_methyltransferases|ERMB	3.522125	0.0109
Tet|tetW_5_AJ427421|Tetracyclines|Tetracycline_resistance_ribosomal_protection_proteins|TETW	3.511888	0.036779
CARD|phgb|AP009048|4303042-4304509|ARO:3003550|mdtP|Multi-drug_resistance|Multi-drug_efflux_pumps|MDTP	3.480891	0.026559
Mdr|AP009048.1|gene3616|Multi-drug_resistance|Multi-drug_efflux_pumps|MDTL	3.478173	0.028324
Bac|AP009048.1|gene3103|Bacitracin|Undecaprenyl_pyrophosphate_phosphatase|BACA	3.445745	0.020148
ACou|NC_012469.1.7686068|Aminocoumarins|Aminocoumarin-resistant_DNA_topoisomerases|PARE|RequiresSNPConfirmation	3.434298	0.000249
CARD|phgb|AP009048|3980025-3981183|ARO:3000795|mdtE|Multi-drug_resistance|Multi-drug_efflux_pumps|MDTE	3.400992	0.029937
Tet|EU434751.1|gene2|Tetracyclines|Tetracycline_resistance_ribosomal_protection_proteins|TETW	3.396945	0.025711
CARD|pvgb|NC_002952|5036-6968|ARO:3003301|Staphylococcus|Aminocoumarins|Aminocoumarin-resistant_DNA_topoisomerases|gyrB|RequiresSNPConfirmation	3.392899	0.015558
222|JQ394987.1|JQ394987|Multi-drug_resistance|Multi-drug_efflux_pumps|MDFA	3.356031	0.025796
CARD|phgb|U00096|4567286-4568519|ARO:3001214|mdtM|Multi-drug_resistance|Multi-drug_efflux_pumps|MDTC	3.319964	0.032239

CARD|phgb|FJ768952|0-1488|ARO:3000237|tolC|Multi-drug_resistance|Multi-drug_efflux_pumps|TOLC	3.31457	0.029937
Flq|CP003275.1|gene5239|Fluoroquinolones|Fluoroquinolone-resistant_DNA_topoisomerases|GYRA|RequiresSNPConfirmation	3.281213	0.006611
Flq|CP000034.1|gene3218|Fluoroquinolones|Fluoroquinolone-resistant_DNA_topoisomerases|PARC|RequiresSNPConfirmation	3.245899	0.009998
1090|DQ303459.3|DQ303459|betalactams|Class_A_betalactamases|CTX	3.238795	0.023877
ACou|NC_007622.3794232|Aminocoumarins|Aminocoumarin-resistant_DNA_topoisomerases|PARE|RequiresSNPConfirmation	3.23569	0.008921
MLS|msrD_3_AF227520|MLS|Macrolide_resistance_efflux_pumps|MSRD	3.165335	0.007355
CARD|phgb|U00096|1124117-1125326|ARO:3001216|mdtH|Multi-drug_resistance|Multi-drug_efflux_pumps|MDTH	3.157944	0.034433
CARD|phgb|D78168|1591-3130|ARO:3000254|emrY|Multi-drug_resistance|Multi-drug_efflux_pumps|EMRY	3.137179	0.040869
Bla|AMPH_Ecoli|AP012030|395554-396711|1158|betalactams|Penicillin_binding_protein|AMPH	3.127537	0.042188
25|JN645706.1|JN645706|betalactams|Penicillin_binding_protein|PBP2X	3.127129	0.003825
CARD|phgb|AP009048|4306556-4307588|ARO:3003548|mdtN|Multi-drug_resistance|Multi-drug_efflux_pumps|MDTN	3.113903	0.043479
CARD|pvgb|NC_002952|1417762-1419760|ARO:3003315|Staphylococcus|Fluoroquinolones|Fluoroquinolone-resistant_DNA_topoisomerases|parE|RequiresSNPConfirmation	3.113369	0.005429
Tet|TetW|AJ222769|3687-5606|1920|Tetracyclines|Tetracycline_resistance_ribosomal_protection_proteins|TETW	3.10601	0.022918
CARD|phgb|U00096|1188315-1189776|ARO:3000835|phoQ|Multi-drug_resistance|MDR_regulator|PHOQ	3.069706	0.043953
CARD|phgb|D78168|536-1592|ARO:3000206|emrK|Multi-drug_resistance|Multi-drug_efflux_pumps|EMRK	3.050985	0.040245
Rif|CP003248.2|gene696|Rifampin|Rifampin-resistant_beta-subunit_of_RNA_polymerase_RpoB|RPOB|RequiresSNPConfirmation	3.013207	0.014656
Fos|fosA_2_AGDM01000012|Fosfomycin|Fosfomycin_thiol_transferases|FOSA	3.001212	0.026297
CARD|pvgb|NC_007779|986315-987404|ARO:3003390|Escherichia|Multi-drug_resistance|MDR_mutant_porin_proteins|OMPF|RequiresSNPConfirmation	2.941379	0.040096
Mdr|AP009048.1|gene3414|Multi-drug_resistance|MDR_regulator|CPXAR	2.936078	0.033647
Tmt|DfrA5|X12868|1306-1779|474|Trimethoprim|Dihydrofolate_reductase|DHFR|RequiresSNPConfirmation	2.872087	0.016082
Flq|NC_002695.1.916822|Fluoroquinolones|Fluoroquinolone-resistant_DNA_topoisomerases|GYRA|RequiresSNPConfirmation	2.855839	0.037008
Mdr|CP000034.1|gene3834|Multi-drug_resistance|MDR_regulator|CPXAR	2.847657	0.043953
ACou|NC_002952.2859942|Aminocoumarins|Aminocoumarin-resistant_DNA_topoisomerases|PARE|RequiresSNPConfirmation	2.747109	0.043934
CARD|phgb|NC_007779|1621287-1621671|ARO:3000263|marA|Multi-drug_resistance|MDR_regulator|MARA	2.666231	0.048077
Mdr|NC_002695.1.914983|Multi-drug_resistance|MDR_regulator|CPXAR	2.665751	0.043109
Mdr|CP000647.1|gene2517|Multi-drug_resistance|MDR_regulator|ASMA	2.662098	0.047145
Flq|NC_002745.1124026|Fluoroquinolones|Fluoroquinolone-resistant_DNA_topoisomerases|PARC|RequiresSNPConfirmation	2.606064	0.043953
gi|41816|emb|X57972.1|betalactams|Class_A_betalactamases|TEM	2.598093	0.036797
CARD|pvgb|AL123456|759806-763325|ARO:3003283|Mycobacterium|Rifampin|Rifampin-resistant_beta-subunit_of_RNA_polymerase_RpoB|RPOB|RequiresSNPConfirmation	2.515975	0.016082
Rif|AE000516.2|gene708|Rifampin|Rifampin-resistant_beta-subunit_of_RNA_polymerase_RpoB|RPOB|RequiresSNPConfirmation	2.36511	0.02685
Phe|CatB4|EU935739|59054-59602|549|Phenicol|Chloramphenicol_acetyltransferases|CATB|RequiresSNPConfirmation	2.285494	0.038788

Differentially abundant genes identified using metagenomic shotgun sequencing (MGS) sequencing between antibiotic treated children and controls are shown here. To validate our Fluidigm results we performed a fitZig (zero-inflated Gaussian mixture model) analysis on the 32 samples obtained at week 1 that were analysed using shotgun metagenomics sequencing. Positive log2 fold change values indicate a gene was more abundant in the antibiotic treated children. The Benjamini-Hochberg method was applied to correct for multiple testing and adjusted p-values (p.adj) are shown.

Supplementary Table 23: List of Fluidigm primers used

AMR gene	Accession number	Forward primer	Reverse primer	Resistance to	Organism
aac(3')-Ii(acde)	HQ246166.1	TGACGTAT GAGATGCC GATG	GAGAATGC CGTTTGAAT CGT	aminoglycosides	*S. flexneri*
aac(6')-aph(2'')	ABY79711.1	TCCAAGAG CAATAAGG GCATA	TGCCCTCG TGTAATTCA TGT	aminoglycosides	***Synthetic M. genitalium JCVI-1.0***<br>*Enterococcus*<br>*Macrococcus*<br>*Staphylococcus*<br>*Streptococcus*
aac(6')-Ib	KM387722.1	TTGCAATG CTGAATGG AGAG	TGGTCTATT CCGCGTAC TCC	aminoglycosides	*S. senftenberg*
aac(6')-Ii	WP_00229398 9.1	AGACAGCT CGGCAGAA GAAG	ACCGTATT GAGGGATT GCAC	aminoglycosides	*E. faecium*
aac(6')-IIa	ACR24243.1	GGAACACT ACCTGCCC AGAG	GCGACGTA CGACTGAG CATA	aminoglycosides	***P. aeruginosa***
aadA	ADW23165.1	CAGCGGAG GAATTCTTT GAC	GCTGCGAG TTCCATAGC TTC	aminoglycosides	***P. aeruginosa***<br>*E. coli*
aadE	CAZ55809.1	TGTGCCGC AAAGAGAT ACTG	AACCTTCCA CGACATCA TCC	aminoglycosides	***S. suis***<br>*S. epidermidis*
aadE-like gene	AAW34138.1	GCATGATT TCCTGGCT GATT	CCACAATTC CTCTGGGA CAT	aminoglycosides	***C. jejuni***<br>*Enterococcus*
acrA	ACI36997.1	GAAGGTAG CGACATCG AAGC	CTTTCGCC AGATCACC TTTC	aminoglycosides and beta-lactams	***E. coli O157:H7***<br>*Citrobacter*<br>*Enterobacter*<br>*Escherichia*<br>*Klebsiella*<br>*Salmonella*<br>*Shigella*
aph(2'')-Ib	AF207840.1	ATCAAATC CCTGCGGT AGTG	CAAGGGCA TCCTTTTCC TTT	aminoglycosides	***E. faecium***<br>*E. coli*
aph(2'')-I(de)	AAC14693.1	CGGAGGTG GTTTTTACA GGA	TTGCTTCG GCAGATTAT TGA	aminoglycosides	***E. casseliflavus***
aph(3')-Ia, -Ic	CAQ58482.1	ATTCTCAC CGGATTCA GTCG	GATTCCGA CTCGTCCA ACAT	aminoglycosides	*E. coli*
aph(3')-III	ACB90577.1	CCGGTATA AAGGGACC ACCT	CTTTGGAA CAGGCAGC TTTC	aminoglycosides	***S. pneumoniae***<br>*Bacillus*<br>*Bacteroides*<br>*Campylobacter*<br>*Enterococcus*<br>*Escherichia*<br>*Roseburia*

					Staphylococcus<br>*Streptococcus*
bla$_{AMPC}$	ABF06289.1	ACCGCTAA ACAGTGGA ATGG	GCAAGTCG CTTGAGGA TTTC	beta-lactams	***S. flexneri***
bla$_{CMY-2}$	AAZ99133.1	CGATCCGG TCACGAAA TACT	CCTGCCGT ATAGGTGG CTAA	beta-lactams	***E. coli***
bla$_{CTX-M}$	ABG46354.1	ACTATGGC ACCACCAA CGAT	GGTTGAGG CTGGGTGA AGTA	beta-lactams	***E. coli***
bla$_{KPC}$	AEL12451.1	TGGCTAAA GGGAAACA CGAC	TAGTCATTT GCCGTGCC ATA	beta-lactams	***P. aeruginosa***<br>*Acinetobacter spp*
bla$_{NDM}$	CAZ39946.1	TGGATCAA GCAGGAGA TCAA	ATTGGCATA AGTCGCAA TCC	beta-lactams	***K. pneumonia***<br>*E. cloacae*<br>*E. coli*<br>*Proteus spp*<br>*C. freundii*<br>*M. morganii*<br>*Providencia spp*<br>*K. oxytoca*
bla$_{OXA}$	AAP70012.1	GTGGCATC GATTATCG GAAT	AGAGCACA ACTACGCC CTGT	beta-lactams	***K. pneumoniae***
bla$_{TEM}$	NP_775035.1	AAGCCATA CCAAACGA CGAG	TTGCCGGG AAGCTAGA GTAA	beta-lactams	***C. freundii***
cblA	AAA66962.1	TGCCTGCG ACATCTTG ATAG	CCGTCTTCT GTTTCCGA GAG	beta-lactams	***B. uniformis***
cfxA	AY769933.1	GCGCAAAT CCTCCTTT AACA	ACAATAACC GCCACACC AAT	beta-lactams	***B. fragilis***
ermB	BAH18720.1	GGTTGCTC TTGCACAC TCAA	CTGTGGTA TGGCGGGT AAGT	macrolides	***M. caseolyticus***
ermC	BAE05991.1	TGAAATCG GCTCAGGA AAAG	GGTCTATTT CAATGGCA GTTACG	macrolides	***S. haemolyticus***
mcr-1	Liu 2016 Lancet Infect Dis	5′- TCGGACTC AAAAGGCG T GAT-3′	5′- GACATCGC GGCATTCG TTAT-3′	colistin	***E.coli***<br>*K. pneumoniae*<br>*P.aeruginosa*
mecA	YP_184944.1	TCCAGGAA TGCAGAAA GACC	GGCCAATT CCACATTGT TTC	beta-lactams/ marker for MRSA	***S. aureus***
spc	AAL05549.1	TGACGAAC GCAATGTG ATTT	TCAGCTGC CAGATCTTT TGA	aminoglycosides	***E. faecalis***
strB	CAJ77026.1	GGCGATTA TAGCCGAT CAAA	CGCGACTG GAGAACAT GATA	aminoglycosides	***A. baumannii***

tetQ	Y08615.1	GCAAAGGA AGGCATAC AAGC	AAACGCTC CAAATTCAC ACC	tetracyclines	***B. fragilis***
vanA	ACP19236.1	GTGCGGTA TTGGGAAA CAGT	TGCGTTTTC AGAGCCTT TTT	vancomycin	***E. faecium***
vanB	WP_03248974 6.1	CCTGCCTG GTTTTACAT CGT	GCTGTCAA TCAGTGCA GGAA	vancomycin	***E. faecalis***
16S rRNA	Gloor 2010 PloS ONE	CAACGCGA RGAACCTT ACC	ACAACACG AGCTGACG AC		

Organisms in bold are listed under the specified accession number in the Antibiotic Resistance Database.
