# Supplementary material for: Effects of early-life antibiotics on the developing infant gut microbiome and resistome: a randomized trial
Source: Nat Commun. 2022 Feb 16;13:893. doi: 10.1038/s41467-022-28525-z (PMC8850541; doi:10.1038/s41467-022-28525-z)
Supplement: Supplementary file 3 — Reporting Summary [file 41467_2022_28525_MOESM3_ESM.pdf]

nature portfolio

Corresponding author(s): Debby Bogaert

Last updated by author(s): Dec 7, 2021

Reporting Summary

Nature Portfolio wishes to improve the reproducibility of the work that we publish. This form provides structure for consistency and transparency in reporting. For further information on Nature Portfolio policies, see our Editorial Policies and the Editorial Policy Checklist.

Statistics

For all statistical analyses, confirm that the following items are present in the figure legend, table legend, main text, or Methods section.

n/a	Confirmed	
☐	☒	The exact sample size (*n*) for each experimental group/condition, given as a discrete number and unit of measurement
☐	☒	A statement on whether measurements were taken from distinct samples or whether the same sample was measured repeatedly
☐	☒	The statistical test(s) used AND whether they are one- or two-sided<br>*Only common tests should be described solely by name; describe more complex techniques in the Methods section.*
☐	☒	A description of all covariates tested
☐	☒	A description of any assumptions or corrections, such as tests of normality and adjustment for multiple comparisons
☐	☒	A full description of the statistical parameters including central tendency (e.g. means) or other basic estimates (e.g. regression coefficient) AND variation (e.g. standard deviation) or associated estimates of uncertainty (e.g. confidence intervals)
☐	☒	For null hypothesis testing, the test statistic (e.g. *F*, *t*, *r*) with confidence intervals, effect sizes, degrees of freedom and *P* value noted<br>*Give P values as exact values whenever suitable.*
☒	☐	For Bayesian analysis, information on the choice of priors and Markov chain Monte Carlo settings
☒	☐	For hierarchical and complex designs, identification of the appropriate level for tests and full reporting of outcomes
☐	☒	Estimates of effect sizes (e.g. Cohen's *d*, Pearson's *r*), indicating how they were calculated

Our web collection on statistics for biologists contains articles on many of the points above.

Software and code

Policy information about availability of computer code

Data collection	Research Manager software (Cloud9 Software BV) was used to collect data for this study.
Data analysis	For the bioinformatic processing and annotation of our 16S rRNA based sequences we used Sickle (version 1.33), BayesHammer (SPAdes genome assembler toolkit, version 3.5.0), PANDAseq (version 2.10), QIIME (version 1.9.1), the Naïve Bayesian RDP classifier (version 2.2) and the SILVA reference database.<br><br>For the bioinformatic processing and annotation of our metagenomic shotgun sequences we used Cutadapt (version cutadapt-1.9.dev2) Bowtie2, the MetaPhlAn2 and MEGARes databases and SAMtools.<br><br>All statistical analyses were performed in R version 3.4.3 within RStudio version 1.1.383 and figures were made using package ggplot2 and ggpubr. R packages used for data analysis were decontam (version 0.99.3), vegan (version 2.4-5) and metagenomeSeq (version 1.20.1).

For manuscripts utilizing custom algorithms or software that are central to the research but not yet described in published literature, software must be made available to editors and reviewers. We strongly encourage code deposition in a community repository (e.g. GitHub). See the Nature Portfolio guidelines for submitting code & software for further information.

Data

Policy information about availability of data

All manuscripts must include a data availability statement. This statement should provide the following information, where applicable:

- Accession codes, unique identifiers, or web links for publicly available datasets
- A description of any restrictions on data availability
- For clinical datasets or third party data, please ensure that the statement adheres to our policy

Sequence data that support the findings of this study have been deposited in the NCBI Sequence Read Archive (SRA) database with BioProject IDs PRJNA481243 (https://www.ncbi.nlm.nih.gov/bioproject/PRJNA481243/), PRJNA524461 (https://www.ncbi.nlm.nih.gov/bioproject/PRJNA524461) and PRJNA555020 (https://www.ncbi.nlm.nih.gov/bioproject/PRJNA555020).

Field-specific reporting

Please select the one below that is the best fit for your research. If you are not sure, read the appropriate sections before making your selection.

- [x] Life sciences
- [ ] Behavioural & social sciences
- [ ] Ecological, evolutionary & environmental sciences

For a reference copy of the document with all sections, see nature.com/documents/nr-reporting-summary-flat.pdf

Life sciences study design

All studies must disclose on these points even when the disclosure is negative.

Sample size	The study was initially powered making use of previously published infant microbiota data, ensuring a power of 0.8 to detect at least significant differences in alpha and beta diversity between groups, and at least two-fold differences in abundance of the 25 most important Operational Taxonomical Units (OTUs). For power calculations, we used data of OTUs with high and low variability and abundance, and varying effect sizes. Our power calculation was verified by the online (HMP-based) tool as soon as this became available. We initially aimed to enrol 132 infants, 44 infants per antibiotic regimen, allowing a drop-out of 10%. Due to the accidental loss of a set of samples from 11 participants, approval was granted by the ethical committee to prolong enrolment in order to replace the lost samples, to ensure power of the study. Eventually, 147 infants were enrolled, 49 per antibiotic regimen.
Data exclusions	From two infants in the amoxicillin + cefotaxime group, no samples were available for analysis, so these infants were excluded from further analyses. Eight (5.4%) children were lost to follow-up due to parents experiencing the collection of samples or completion of questionnaires as too burdensome, or moving abroad.
Replication	In our randomized trial we did not perform laboratory experiments to be replicated. We did, however, in our statistical analyses use both parametric and non-parametric methodology to address our research questions in order to validate our findings and minimize false negative results.
Randomization	Infants were randomly allocated 1:1:1 to three most commonly prescribed intravenous antibiotic combinations for suspected early-onset neonatal sepsis (sEONS) in the Netherlands, namely penicillin + gentamicin, co-amoxiclav + gentamicin or amoxicillin + cefotaxime. The sequence with which participants were allocated to the groups was generated with the Research Manager software (Cloud9 Software BV) by an unaffiliated research nurse using 11 blocks of 12 (4:4:4) and one block of 15 (5:5:5) to enable a balanced randomisation over the three regimens in the four hospitals. Allocation concealment was achieved using sealed, opaque envelopes which were delivered to the hospitals.
Blinding	Blinding was not performed, as this was not deemed relevant in our observational study with shifts in gut microbiome and antimicrobial resistance (AMR) gene composition as primary study outcomes. Also, due to clinical, practical and safety reasons we could not perform blinding.

Reporting for specific materials, systems and methods

We require information from authors about some types of materials, experimental systems and methods used in many studies. Here, indicate whether each material, system or method listed is relevant to your study. If you are not sure if a list item applies to your research, read the appropriate section before selecting a response.

Materials & experimental systems

n/a	Involved in the study
☒	☐ Antibodies
☒	☐ Eukaryotic cell lines
☒	☐ Palaeontology and archaeology
☒	☐ Animals and other organisms
☐	☒ Human research participants
☐	☒ Clinical data
☒	☐ Dual use research of concern

Methods

n/a	Involved in the study
☒	☐ ChIP-seq
☒	☐ Flow cytometry
☒	☐ MRI-based neuroimaging

Human research participants

Policy information about studies involving human research participants

Population characteristics	We performed a randomized study in 147 infants who required broad-spectrum antibiotics for treatment of sEONS in their first week of life. Infants were recruited at the Spaarne Gasthuis Hoofddorp and Haarlem, Diakonessenhuis Utrecht and Tergooiziekenhuis Blaricum in the Netherlands (Zuigelingen En Bacteriële Resistentie na Antibiotica, ZEBRA trial). Inclusion criteria were indication for broad-spectrum antibiotic treatment due to sEONS in the first seven days of life, birth by vaginal delivery or secondary caesarean section (SCS), gestational age of ≥36 weeks, absence of prenatally established underlying morbidity, parental age of ≥18 years and the ability of parents to understand the Dutch or English language. A subset of healthy, term born infants from the Dutch Microbiome Utrecht Infant Study, served as controls. We included 80 out of 120 infants from this birth cohort that were born vaginally or by SCS, had not received antibiotics in the first week of life and whose samples could be age-matched to those of the sEONS infants. Relevant population characteristics of our participants include gender, mode of delivery, exposure to antepartum maternal antibiotics, season of birth, gravidity of mothers, gestational age, birth weight, duration of ruptured membranes, Apgar score, duration of antibiotic treatment, hospital stay duration after birth, siblings, pets, inhouse smoking, education of parents, feeding type, pacifier use, daycare attendance and antibiotic use during follow-up.
Recruitment	Initial enrolment was performed by trained physicians who assigned the groups through selecting one of the sealed randomization envelopes. Neither the research nurse who generated the allocation sequence nor the enrolling physicians were involved with the rest of the trial. A potential selection bias may have occurred as a consequence of an above average number of parents with a higher level of education having opted to participate in our study. This may also have affected the low percentage of inhouse smoking observed, and may therefore affect the generalizability of our results to some extent. Finally, we cannot rule out that parents of sEONS infants were more aware of possible side-effects of antibiotic treatment due to the participation in a trial studying precisely these effects, and therefore this may have affected their hesitance for antibiotic treatment at a later stage.
Ethics oversight	Dutch National Ethics Committee

Note that full information on the approval of the study protocol must also be provided in the manuscript.

Clinical data

Policy information about clinical studies

All manuscripts should comply with the ICMJEguidelines for publication of clinical research and a completedCONSORT checklist must be included with all submissions.

Clinical trial registration	Netherlands Trial Registry NL4882 (ZEBRA) and NL3821 (MUIS).
Study protocol	The study protocol will be shared on request.
Data collection	We followed 147 neonates born ≥36 weeks of gestational age, who were recruited in the period from 16 January 2015 to 13 September 2016 in three different Dutch hospitals (Spaarne Gasthuis Hoofddorp and Haarlem, Diakonessenhuis Utrecht and Tergooiziekenhuis Blaricum), until their first birthday. After discharge from hospital, home visits took place.
Outcomes	The primary outcomes were shifts in gut microbiome and antimicrobial resistance gene composition following antibiotic treatment, compared to controls. A statistical analysis scheme showing the flow in and order of analyses to address the primary research questions can be found in the Supplementary Information. Furthermore, we confirmed our 16S rRNA based sequencing and targeted qPCR results by metagenomic shotgun sequencing of a subset of samples. Also, we correlated OTU and AMR gene abundance.
